# Community Paramedicine Program in Social Housing and Health Service Utilization: A Cluster Randomized Clinical Trial

**DOI:** 10.1001/jamanetworkopen.2024.41288

**Published:** 2024-10-28

**Authors:** Gina Agarwal, Melissa Pirrie, Ricardo Angeles, Francine Marzanek, J. Michael Paterson, Francis Nguyen, Lehana Thabane

**Affiliations:** 1 Department of Family Medicine, McMaster University, Hamilton, Ontario, Canada; 2 Health Research Methods, Evidence, and Impact, Hamilton, Ontario, Canada; 3 ICES, Toronto, Ontario, Canada; 4 St Joseph’s Healthcare Hamilton, Hamilton, Ontario, Canada

## Abstract

**Importance:**

Community Paramedicine at Clinic (CP@clinic) is a chronic disease prevention program that decreases 911 calls for emergency medical services, but its wider system effects are unknown.

**Objective:**

To evaluate the effects of CP@clinic vs usual care on individual-level health service utilization outcomes.

**Design, Setting, and Participants:**

This open-label, pragmatic cluster randomized clinical trial evaluated all residents 55 years or older in 30 social housing buildings in Ontario, Canada, that had (1) a unique postal code, (2) at least 50 apartments, (3) 60% or more residents 55 years or older, and (4) a similar building for pairing (15 intervention and 15 control buildings, pair-matched randomization). The 12-month intervention had a staggered start date from January 1, 2015, to December 1, 2015, and ended between December 31, 2015, and November 30, 2016. Administrative health data analysis was conducted in May 2022.

**Intervention:**

CP@clinic was a health promotion and disease prevention program led by specially trained community paramedics who held weekly drop-in sessions in social housing buildings. These paramedics conducted 1-on-1 risk assessments, provided health education and referrals to relevant community resources, and, with consent, sent assessments to family physicians. Control buildings received usual care (universal health care, including free primary and specialty medical care).

**Main Outcome and Measures:**

Individual-level health service utilization was measured from administrative health data, with ED visits via ambulance as the primary outcome; secondary outcomes included ED visits for any reason, primary care visits, hospitalizations, length of hospital stay, laboratory tests, receipt of home care, transfer to long-term care, and medication initiation. Generalized estimating equations were used to estimate intervention effects on individual-level health service utilization, accounting for trial design and individual-level baselines.

**Results:**

The 30 social housing buildings had 3695 residents (1846 control and 1849 intervention participants; mean [SD] age, 72.8 [9.1] years; 2400 [65.0%] female). Intention-to-treat analysis found no significant difference in ED visits by ambulance (445 of 1849 [24.1%] vs 463 of 1846 [25.1%]; adjusted odds ratio [AOR], 0.97; 95% CI, 0.89-1.05) but found higher antihypertensive medication initiation (74 of 500 [14.8%] vs 47 of 552 [8.5%]; AOR, 1.74; 95% CI, 1.19-2.53) and lower anticoagulant initiation (48 of 1481 [3.2%] vs 69 of 1442 [4.8%]; AOR, 0.68; 95% CI, 0.53-0.86) in the intervention arm vs the control arm. CP@clinic attendance was associated with higher incidence of primary care visits (adjusted incidence rate ratio, 1.10; 95% CI, 1.03-1.17), higher odds of receiving home care (AOR, 1.07; 95% CI, 1.01-1.13), and lower odds of long-term care transfers (AOR, 0.32; 95% CI, 0.13-0.81).

**Conclusions and Relevance:**

In this cluster randomized clinical trial of CP@clinic, the intervention did not affect the rate of ED visits by ambulance; however, there were increased primary care visits and connections to home care services, which may have increased antihypertensive medication initiation and reduced long-term care transfers from social housing. Health policymakers should consider CP@clinic’s impact as an upstream approach to improve care for older adults with low income.

**Trial Registration:**

ClinicalTrials.gov Identifier: NCT02152891

## Key Points

**Question:**

What are the effects of the Community Paramedicine at Clinic (CP@clinic) program on health care utilization compared with usual care?

**Findings:**

In a cluster randomized clinical trial of 3695 residents aged 55 years or older in 30 social housing buildings, the rates of emergency department (ED) visits by ambulance (the primary outcome) were similar between control and intervention buildings; however the intervention group had higher odds of antihypertensive medication initiation, a higher rate of primary care visits, greater odds of receiving home care services, and lower odds of transferring to long-term care during the 12-month intervention, all statistically significant changes.

**Meaning:**

While there was no significant difference in the primary outcome of ED visits by ambulance, CP@clinic did increase primary care visits and connections to home care services; this may have increased antihypertensive medication initiation and reduced long-term care transfers from social housing.

## Teaser

This randomized clinical trial of social housing building residents in Ontario, Canada, evaluates the effects of a community paramedicine program vs usual care on health service use.

## Introduction

Older adults are a rapidly increasing segment of the Canadian population.^1^ Low-income older adults experience the highest rates of chronic diseases and multimorbidity,^2,3,4^ lower health-related quality of life,^5^ and greater mortality rates^6^ compared with other older adults. Consequently, they account for one-third of emergency medical services (EMS) calls,^4,7,8^ some of which are preventable (eg, chronic disease and falls management).^9^ Community health promotion programs targeting low-income older adults have been proposed to address health inequities.^10^

The Community Paramedicine at Clinic (CP@clinic) program is an innovative community health program evaluated using a randomized clinical trial (RCT). CP@clinic led to 0.90 fewer calls per month per 100 apartment units in 5 communities in Ontario, Canada, and has demonstrated improved participant health-related quality of life and reduced chronic disease risk.^11,12^ A subsequent economic analysis found that CP@clinic was a cost-effective solution for the emergency health care system, resulting in a net resource gain,^13^ and many paramedic services have since adopted the program.^14^ However, CP@clinic’s effect on broader health service utilization has not been evaluated.

The objective of this multisite cluster RCT was to evaluate the effects of the CP@clinic program vs usual care on health service utilization outcomes. Universal health care is the default usual care in Ontario, with free primary and specialty medical health care provided to all ages. We hypothesized that health care utilization would be affected by the CP@clinic program. Because the CP@clinic intervention was a drop-in program open to all residents, building-level cluster randomization was the most appropriate design.^15^ These results provide an understanding of CP@clinic’s effect on the broader health system in Ontario and establish an evidence base to drive health system change as community-based, paramedicine-led health promotion programs become standard.^16,17^

## Methods

### Study Design

The study was an open-label cluster RCT randomized at the social housing building level in 30 buildings across 5 Ontario communities. These apartment buildings were for low-income seniors; and rent was capped at 30% of household income, with the remainder subsidized by the government. Details of the methods and intervention have been published elsewhere.^11,12^ This study was approved by the Hamilton Integrated Research Ethics Board, which granted a waiver of consent for cluster-level cohort creation at ICES (formerly known as the Institute for Clinical Evaluative Sciences). Those attending the CP@clinic program (attendees) signed written, informed consent forms to have their CP@clinic assessment data sent to ICES for linkage with their administrative health data at ICES. ICES is an independent, nonprofit research institute whose legal status under Ontario’s health information privacy law allows it to collect and analyze health care and demographic data, without consent, for health system evaluation and improvement. CP@clinic participant data were probabilistically linked to their corresponding health care records at ICES according to name, sex, date of birth, and postal code; Ontario Health Insurance Plan (OHIP) numbers were not collected from participants. This report follows the Consolidated Standards of Reporting Trials (CONSORT) reporting guideline. The trial protocol can be found in Supplement 1.

### Intervention

The intervention was a community paramedicine health promotion and disease prevention program: CP@clinic. Briefly, each week, 2 specially trained paramedics provided a half-day voluntary drop-in session for the social housing building residents in a common space (eg, wellness room) of each building. The community paramedics met with residents one on one to conduct validated health risk assessments (eg, physical measures, cardiometabolic risk screening questionnaires, and tools for assessing social determinants of health). On the basis of the individual’s risk profile, the paramedic worked with the resident to identify which risk factor(s) they wanted to focus on first (eg, low physical activity), provided tailored health education on lifestyle modifications, assisted in goal setting, and provided referrals to relevant community resources (eg, local walking programs, homecare services, and mobility device assessment). Residents were also encouraged to return to the CP@clinic program to discuss their progress and challenges, which allowed the community paramedic to assist in troubleshooting (eg, finding a different resource that may be a better fit), provide encouragement, and select another risk factor that the participant wanted to address next. For those who had a family physician and consented, assessment results were sent to the physician. The intervention had a staggered start date (between January 1 and December 1, 2015) and ran for 12 months (between December 31, 2015, and November 30, 2016, respectively).

### Study Participants 

Because the intervention was available to all building residents, the unit of randomization was the building. The building-level inclusion criteria were as follows: (1) a unique postal code not shared with neighboring residences, (2) at least 50 apartment units, (3) at least 60% of residents aged 55 years or older, and (4) presence of another building of similar size, demographic characteristics, and geographic location to permit paired randomization. There were no exclusion criteria.

The individual-level, closed cohort included all building residents 55 years or older who lived at the study postal codes in the quarter immediately preceding the intervention start date according to the OHIP Registered Persons Database (RPDB). All building residents were permitted to participate in the CP@clinic program regardless of age, but only those who met the age criterion were included in the analysis. Individuals were excluded from analysis if they were 120 years or older, ineligible for OHIP benefits, or had no encounters with the health care system in the preceding 7 years.

### Randomization and Masking

Buildings were paired based on similar characteristics, such as location, number of units, percentage of seniors, previous year EMS call rate, and level of programming, in consultation with the municipal housing providers (see prior publication^12^ for a description of the matching process). Using a computer-generated randomization program (Research Randomizer; Social Psychology Network), the research team randomly allocated 1 building from each pair to receive the intervention (CP@clinic) and 1 building to usual care. Masking of intervention building residents was not feasible due to it being a drop-in program in a common space, although residents were not informed of the precise details of the trial outcome measures. In addition, control building residents were not informed they were study controls and would have only known about the intervention if made aware by intervention building residents or local media.

### Procedures

The social housing building postal codes were sent to ICES for the building-level cohort creation for the intention-to-treat analysis. Demographic characteristics, health status profile, and health system utilization outcomes were obtained from multiple ICES-held datasets and ICES-derived cohorts, linked using unique encoded identifiers. For attendees, personal identifiers collected from the CP@clinic database were securely transmitted to ICES for linkage to facilitate the sensitivity analyses. All outcome data in this study are from administrative health datasets. The study team led the data analysis conducted by an ICES analyst to maintain privacy.

#### Demographic Characteristics and Health Status

Participant age and sex at the intervention start date were obtained from the OHIP RPDB; data on race and ethnicity were not available. Validated case definitions identified whether participants had 5 chronic diseases reliably captured in administrative health data: diabetes,^18^ congestive heart failure,^19^ chronic obstructive pulmonary disease,^20^ hypertension,^21^ and dementia.^22^ Cardiovascular disease and peripheral vascular disease status were determined using a 3-year lookback from the intervention start date based on physician service claims, emergency department (ED) records, and hospital discharge abstracts. Details are available in eTable 1 in Supplement 2. The Johns Hopkins ACG System, version 10 was used to quantify multimorbidity (number of aggregated diagnosis groups) and determine participants flagged as frail.^23,24^

#### Health Service Utilization Outcomes

For the 12-month preintervention and intervention periods, all unplanned ED visits for any cause and unplanned ED visits via ambulance for any cause were obtained from the Canadian Institute for Health Information (CIHI) National Ambulatory Care Reporting System. Each outcome was dichotomized as having at least 1 ED visit vs none. Nonelective hospital admissions for any cause (ie, urgent or emergent) were obtained from the CIHI Discharge Abstract Database using an episode-of-care approach treating transfers among hospitals within 24 hours as a single event. Total length of stay for the episode of care was computed as the sum of days hospitalized.

Primary care visits during the 12-month preintervention and intervention periods were obtained from OHIP physician billings, capturing outpatient visits where the physician’s main specialty was family physician or general practitioner in the ICES Physician Database. Multiple billings from the same physician on the same date were considered a single visit. Diabetes, kidney function, and lipid laboratory tests were obtained from the Ontario Laboratories Information System (see eTable 1 in Supplement 2 for details).

Prescription drug claim data were available for those enrolled in the Ontario Drug Benefit (ODB) Plan, which covers individuals (1) receiving government financial benefits (eg, Ontario Works, Ontario Disability Support Program), (2) enrolled in the Trillium Drug Program (for those with very high prescription drug costs relative to income), or (3) aged 65 years or older. We grouped drug claims into 4 categories: diabetes, antihypertensives, anticoagulants, and statins or fibrates. New medication initiation was defined as having a medication initiated during the 12-month intervention period and having no prior ODB claim for medications within the same category during the 12 months before the intervention. Thus, individuals needed to have at least 1 year of medication data available before the intervention to be included. See eTable 2 in Supplement 1 for medications within each drug category.

Building residents with at least 1 visit from a home and community support service provider in the Ontario Homecare Database were classified as receiving homecare services during the 12-month preintervention or intervention period. Finally, transfers to long-term care (LTC) for short or long stays were defined by having at least 1 OHIP physician bill with an LTC-specific fee code or institution type indicating the service was provided within LTC during the intervention period.

### Outcome Measures

The primary outcome was individual-level ED visits via ambulance as a main measure of health care utilization. Secondary outcomes included any ED visits, physician visits (primary care), hospitalizations, length of hospital stay, laboratory tests, receipt of home care, transfer to LTC, and prescription medication initiation among those with provincial drug benefits. Each of these individual-level outcomes was assessed through the ICES administrative datasets for 12 months during the intervention period (see the Procedures section for outcome definitions and details of data sources for each outcome).

### Statistical Analysis

Data analysis was conducted in May 2022. Sample size was calculated based on the CP@clinic pilot^3^ in the same population, which reported a 25% decrease in EMS calls during the intervention year compared with the previous 2 years.^3^ Due to there being no other readily available data in this setting, we assumed a similar effect size in ED visits by ambulance adjusted for baseline and conservatively estimated a 15% effect size with an intracluster correlation coefficient (ICC) of 0.07. Using standard parameters (statistical power = 0.80, α = .05), the required sample size was a minimum of 1108 participants (11 buildings with 100 units) in each trial arm. All analyses were conducted using individual record–level data and 2-sided analyses and using a threshold of significance of *P* < .05. Demographic and health status characteristics were described using descriptive statistics.

**Figure 1.**
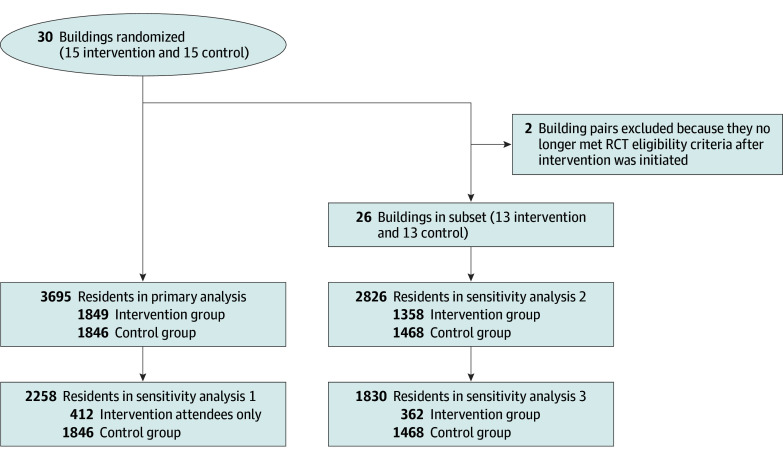
Community Paramedicine at Clinic (CP@clinic) Randomized Clinical Trial (RCT) Profile

For the study outcomes, descriptive analyses were performed for the 12-month preintervention period and the 12-month intervention period. The intervention start date was staggered, so the periods were relative and specific to each set of pair-randomized buildings. For the intention-to-treat analysis, we used generalized estimating equation models assuming an independent correlation matrix to estimate the individual-level effects of the CP@clinic intervention vs usual care, while accounting for clustering of residents within buildings and adjusting for the paired building trial design and individual-level baseline (ie, each resident’s preintervention year measure). For the transfers to LTC outcome, there was no adjustment for baseline because most residents who moved to LTC in the preintervention year were expected to no longer be residing at the building. A sensitivity analysis limited the modeling to the subset of intervention building residents who had attended at least 1 CP@clinic program session compared with control building residents. During the intervention, it was discovered that 2 buildings did not meet the eligibility criteria; this circumstance has been described in detail elsewhere.^12^ Therefore, further sensitivity analyses were conducted using the same methods but excluding these 2 pairs (Figure 1). The results are reported as adjusted odds ratio (AOR) for binary outcomes and adjusted incidence rate ratio (AIRR) for counts, with corresponding 95% CIs. All descriptive and generalized estimating equation analyses were performed using SAS software, version 9.4 (SAS Institute Inc).

## Results

### Participant Characteristics

Per the RCT protocol,^25^ 30 buildings were pair-randomized with 15 assigned to intervention and 15 to control (see Figure 1 for overview of study flow and participant numbers). Of the 3695 residents in the primary analysis (Figure 1), the success rate in linking individuals with their corresponding OHIP RPDB record was 92%. Cluster size ranged from 40 to 391 across the 30 sites. The primary outcome ICC was 0.004 and the secondary outcome ICCs ranged from −0.012 to 0.039.

The mean (SD) participant age was 72.8 (9.1) years; 2400 participants (65.0%) were female and 1295 (35.0%) were male; and 3482 (94.2%) were ODB eligible (eTable 3 in Supplement 2). The intervention and control groups had similar rates of most chronic diseases (Table 1).

**Table 1.** Participant Characteristics and Baseline Health Status of Intervention Building Residents, Control Building Residents, and Intervention Attendees

Characteristic	No. (%) of residents^a^					
	All buildings (N = 30)			Subset of buildings (n = 26)		
	Control, all residents (n = 1846)	Intervention		Control, all residents (n = 1468)	Intervention	
		All residents (n = 1849)	Attendees only (n = 412)		All residents (n = 1358)	Attendees only (n = 362)
Demographics						
Age, y						
Mean (SD)	72.5 (9.0)	73.1 (9.1)	73.5 (8.9)	73.6 (8.9)	73.7 (8.4)	74.1 (8.6)
Median (IQR)	72 (65-79)	73 (66-79)	74 (67-80)	73 (67-80)	74 (67-79)	74 (67-80)
Sex						
Female	1148 (62.2)	1252 (67.7)	339 (82.3)	963 (65.6)	981 (72.2)	302 (83.4)
Male	698 (37.8)	597 (32.3)	73 (17.7)	505 (34.4)	377 (27.8)	60 (16.6)
Health status						
Diabetes	637 (34.5)	637 (34.5)	150 (36.4)	515 (35.1)	469 (34.5)	125 (34.5)
COPD	722 (39.1)	603 (32.6)	168 (40.8)	568 (38.7)	437 (32.2)	145 (40.1)
Congestive heart failure	242 (13.1)	223 (12.1)	54 (13.1)	214 (14.6)	147 (10.8)	45 (12.4)
Hypertension	1269 (68.7)	1326 (71.7)	311 (75.5)	1041 (70.9)	1005 (74.0)	277 (76.5)
Dementia	118 (6.4)	132 (7.1)	29 (7.0)	102 (6.9)	89 (6.6)	25 (6.9)
Cardiovascular disease	104 (5.6)	105 (5.7)	19 (4.6)	87 (5.9)	77 (5.7)	18 (5.0)
Frailty^b^	310 (16.8)	301 (16.3)	67 (16.3)	266 (18.1)	201 (14.8)	56 (15.5)
Multimorbidity, No. of ADGs						
0-4	304 (16.5)	263 (14.2)	28 (6.8)	226 (15.4)	185 (13.6)	24 (6.6)
5-9	631 (34.2)	614 (33.2)	116 (28.2)	494 (33.7)	450 (33.1)	103 (28.5)
≥10	911 (49.3)	972 (52.6)	268 (65.0)	748 (51.0)	723 (53.2)	235 (64.9)

Abbreviations: ADGs, aggregated diagnosis groups (from the ACG System); COPD, chronic obstructive pulmonary disease.

^a^ Unless otherwise indicated.

^b^ Frailty flag from the Johns Hopkins ACG System.

Three sensitivity analyses were conducted: those who attended the program (n = 412) vs controls (n = 1846), all residents excluding 2 building pairs not meeting eligibility criteria (n = 1358 intervention building residents and n = 1468 controls), and combining the first 2 conditions with attendees (n = 362) compared with controls (n = 1468) (Figure 1). Attendees (n = 412) were predominantly female (339 [82.3%]), had greater multimorbidity (≥10 aggregated diagnosis groups, 268 [65.0%] vs 911 [49.3%]), and were more likely to have hypertension at baseline (311 [75.5%] vs 1269 [68.7%]) compared with controls (Table 1).

### Health Service Utilization

#### Intention-to-Treat Analysis

The primary outcome comparing ED visits via ambulance in intervention building residents (attendees and nonattendees) vs control buildings demonstrated no significant difference (445 [24.1%] vs 463 [25.1%]; AOR, 0.97; 95% CI, 0.89-1.05) (Table 2). The secondary outcome analysis found that among residents who were ODB eligible, those in the intervention group had significantly higher odds of antihypertensive medication initiation (74 of 500 [14.8%] vs 47 of 552 [8.5%]; AOR, 1.74; 95% CI, 1.19-2.53) and lower odds of anticoagulant initiation (48 of 1481 [3.2%] vs 69 of 1442 [4.8%]; AOR, 0.68; 95% CI, 0.53-0.86) (eTable 4 in Supplement 2). For all other secondary outcomes, there was no significant difference observed (Table 2 and eTable 4 in Supplement 2).

**Table 2.** Intention-to-Treat Analysis of Health Care Utilization Outcomes, Except Medication Initiation, for All Intervention and Control Building Residents

Outcome	No. (%) of residents^a^				AOR (95% CI)^b^
	Baseline (N = 30 buildings) for all residents		Postintervention (N = 30 buildings) for all residents		
	Control (n = 1846)	Intervention (n = 1849)	Control (n = 1846)	Intervention (n = 1849)	
Binary outcomes					
ED visits	689 (37.3)	738 (39.9)	764 (41.4)	782 (42.3)	0.99 (0.93-1.06)
ED visits by ambulance	368 (19.9)	368 (19.9)	463 (25.1)	445 (24.1)	0.97 (0.89-1.05)
Hospital admissions	264 (14.3)	268 (14.5)	338 (18.3)	326 (17.6)	0.98 (0.88-1.09)
Home care services	430 (23.3)	449 (24.3)	499 (27.0)	511 (27.6)	0.99 (0.92-1.07)
Transfers to long-term care	NA	NA	83 (4.5)	75 (4.1)	0.90 (0.68-1.20)
Kidney function laboratory test	1229 (66.6)	1302 (70.4)	1277 (69.2)	1324 (71.6)	0.99 (0.95-1.04)
Electrolyte laboratory test	1071 (58.0)	1136 (61.4)	1095 (59.3)	1149 (62.1)	1.01 (0.95-1.07)
Lipids laboratory test	1011 (54.8)	1047 (56.6)	999 (54.1)	1055 (57.1)	1.02 (0.98-1.08)
Diabetes laboratory test	946 (51.2)	977 (52.8)	1000 (54.2)	1043 (56.4)	1.01 (0.97-1.05)
Continuous outcomes					
No. of primary care visits, mean (SD)	5.94 (6.59)	6.04 (6.27)	6.29 (6.77)	6.22 (6.72)	AIRR, 1.00 (0.94-1.05)
Hospital length of stay, mean (SD), d^c^	13.05 (21.89)	12.16 (20.35)	18.78 (37.20)	17.93 (25.44)	AIRR, 0.94 (0.83-1.07)

Abbreviations: AIRR, adjusted incidence rate ratio; AOR, adjusted odds ratio; ED, emergency department; NA, not applicable.

^a^ Unless otherwise indicated.

^b^ Except for the transfers to long-term care, all AORs and AIRRs were calculated using generalized estimated equation models adjusted for clustering of residents within buildings, building pairing (trial design), and the baseline value. For the transfers to long-term care outcome, the generalized estimated equation model was adjusted for clustering of residents within buildings and the building pairing only.

^c^ The hospital length of stay outcome was restricted to only those with a hospital admission.

#### Sensitivity Analysis 1

**Figure 2.**
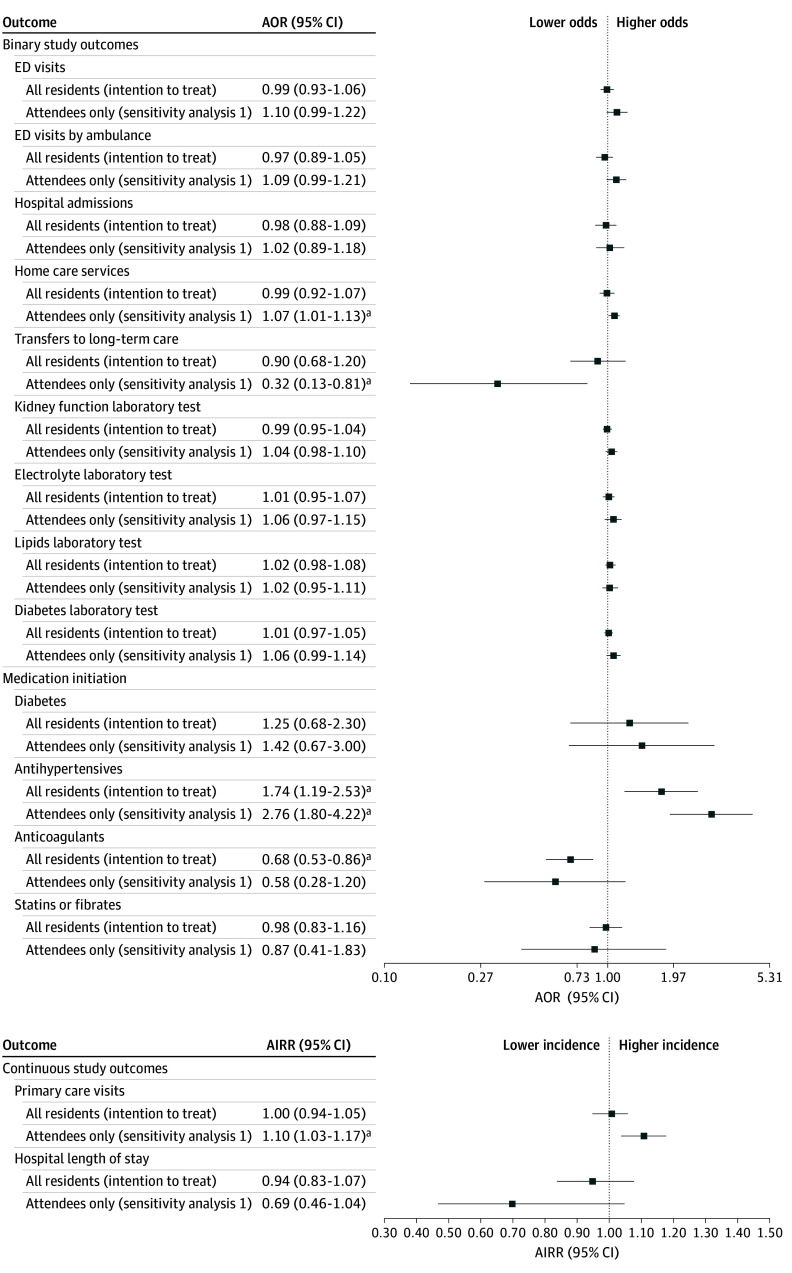
Effect of Community Paramedicine at Clinic (CP@clinic) on Health Care Utilization Outcomes Over 12 Months in the Full Sample of 30 Study Buildings AIRR indicates adjusted incident rate ratio; AOR, adjusted odds ratio; and ED, emergency department. ^a^Significant at *P* < .05.

For those who attended the intervention compared with control building residents (Figure 2; eTable 5 in Supplement 2), no significant difference was observed in the primary outcome of ED visits via ambulance (AOR, 1.09; 95% CI, 0.99-1.21). However, for the secondary outcomes, attending the CP@clinic program was associated with a significantly higher incidence of primary care visits (AIRR, 1.10; 95% CI, 1.03-1.17), higher odds of receiving home care services (AOR, 1.07; 95% CI, 1.01-1.13), and lower odds of LTC transfers (AOR, 0.32; 95% CI, 0.13-0.81). Among those eligible for ODB, antihypertensive medications had significantly higher odds of being initiated (AOR, 2.76; 95% CI, 1.80-4.22). In addition, there were lower odds of anticoagulant initiation in the intervention attendees (AOR, 0.58; 95% CI, 0.28-1.20), but this finding was not statistically significant. For the remaining secondary outcomes, no significant differences were observed.

#### Sensitivity Analysis 2

**Figure 3.**
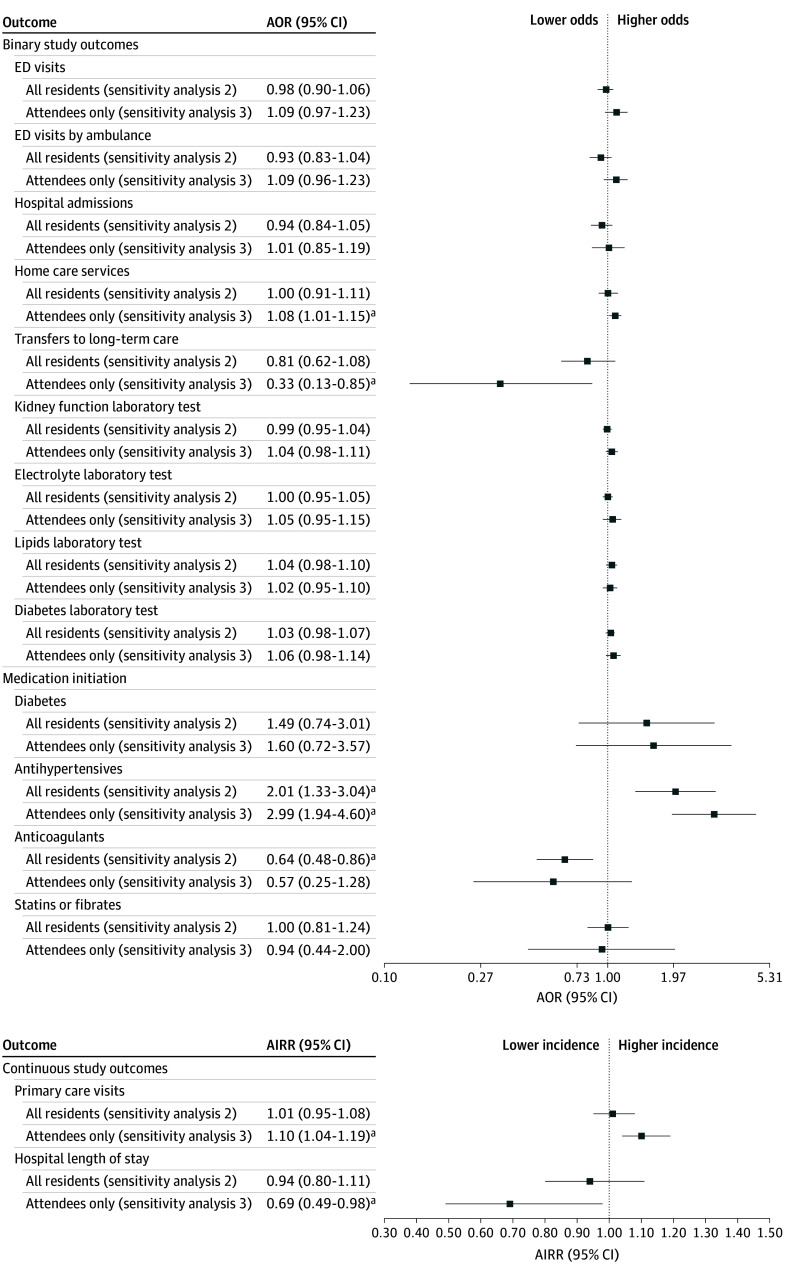
Effect of Community Paramedicine at Clinic (CP@clinic) on Health Care Utilization Outcomes Over 12 Months in the Subset of 26 Study Buildings In the subset of study buildings (n = 26), 2 pairs were removed that no longer met eligibility criteria after intervention began. AIRR indicates adjusted incident rate ratio; AOR, adjusted odds ratio; and ED, emergency department. ^a^Significant at *P* < .05.

When 2 building pairs were excluded due to their eligibility changing during the RCT, the cohort descriptive analysis comparing building residents (both attendees and nonattendees) with the control buildings found no significant difference in the primary outcome (AOR, 0.93; 95% CI, 0.83-1.04) (Figure 3; eTable 6 in Supplement 2). For the secondary outcomes, there were significantly higher odds of antihypertensive medication initiation (AOR, 2.01; 95% CI, 1.33-3.04) and lower odds of anticoagulant medication initiation (AOR, 0.64; 95% CI, 0.48-0.86). No significant associations were observed in the remaining secondary outcomes.

#### Sensitivity Analysis 3

When comparing those who attended the intervention with the control building residents, with the 2 building pairs excluded, no significant difference was found in the primary outcome (AOR, 1.09; 95% CI, 0.96-1.23) (Figure 3; eTable 7 in Supplement 2). For the secondary outcomes, CP@clinic attendance was associated with a significantly higher incidence rate of primary care visits (AIRR, 1.10; 95% CI, 1.04-1.19), lower incidence rate for length of hospital stay (AIRR, 0.69; 95% CI, 0.49-0.98), lower odds of LTC transfers (AOR, 0.33; 95% CI, 0.13-0.85), and higher odds of home care services (AOR, 1.08; 95% CI, 1.01-1.15). Among those eligible for ODB, antihypertensive medications had significantly higher odds of being initiated (AOR, 2.99; 95% CI, 1.94-4.60). Odds of anticoagulant initiation were lower in CP@clinic attendees (AOR, 0.57; 95% CI, 0.25-1.28), but this finding was not significant. For all other secondary outcomes analyzed, no significant differences were observed.

## Discussion

This multisite cluster RCT evaluated the effects of the CP@clinic program vs usual care on health service utilization outcomes. Our intention-to-treat analysis found that although residents of intervention buildings did not have significant differences in ED visits by ambulance, there was a significantly higher rate of antihypertensive medication initiation. This finding implies that uncontrolled or undiagnosed hypertension was being addressed by offering CP@clinic sessions in the social housing buildings, most likely through referrals to their primary care physicians. A qualitative study found that CP@clinic attendees who were reluctant to seek preventive medical care were more willing when it was recommended by the community paramedics, whom they highly respected.^26^ In addition, the relationship between medication initiation and primary care visits may account for the higher incidence rate of primary care visits for attendees vs nonattendees. This finding was consistent across sensitivity analyses. Hypertension is a risk factor for several cardiovascular diseases,^27^ and appropriate control in primary care is imperative for cardiac risk reduction.^27^

Unexpectedly, the rate of ED visits by ambulance was not significantly different between study groups. Previously, an RCT found that EMS calls significantly decreased in intervention buildings where CP@clinic was implemented^12^; therefore, a decrease in the rate of ED visits by ambulance was hypothesized. However, EMS calls and ED visits are vastly different, and the former may not always result in an ED visit. Indeed, the novel effect of the CP@clinic program on the health care system is that it reduces EMS demand for low-acuity conditions but not necessarily ED visits. Therefore, for paramedic services, the value in implementing the CP@clinic program remains because it reduces those low-acuity EMS calls for which transport is not necessary.

A common issue faced by community health programs is low participation rates, especially from marginalized populations,^28,29,30^ but, as previously reported, CP@clinic had a mean participation rate of 39%, and more than 80% of participants attended the program at least 3 times during the RCT.^12^ Our cohort descriptive analyses found that attendees had higher rates of multiple chronic diseases. Therefore, the population in greatest need of CP@clinic was receiving it appropriately. As a result of program attendance, attendees also had higher rates of primary care visits, hypertension medication initiation, and home care services. Conversely, attendees had fewer LTC transfers and fewer days in the hospital when admitted. In a qualitative study that interviewed CP@clinic attendees, attendees viewed the community paramedic role as being highly trusted professionals who advocated for their well-being and connected them with resources.^26^ This finding aligns with the present study’s findings that attendees had more homecare and fewer transfers to LTC in the administrative records. Therefore, all analyses imply that CP@clinic may increase attendees’ connections to important health care services, thereby averting health crises.

Community paramedicine interventions are rarely linked to outcomes from administrative data using RCT methods. A 2022 literature review identified 21 articles examining health system outcomes, of which 18 were peer reviewed, including 4 reporting RCTs (3 for the same trial) and 14 using administrative data.^31^ All administrative data studies in this review used observational methods, and community paramedicine was hypothesized as a potential reason for improved outcomes.^32^ Of the 2 RCTs, 1 was the trial currently being reported in this article,^12^ and the other, from the UK,^33,34^ examined paramedic practitioners as an alternate dispatch response. Therefore, our trial is, to our knowledge, the only cluster RCT to study a paramedic wellness program that did not require an emergency call to be initiated.

### Strengths and Limitations

This study has several strengths, including the robust, pragmatic cluster randomization design and use of administrative data, which were not reliant on self-report or recall. Community programs are rarely evaluated using cluster RCT methods and administrative data because both are hard to execute in a pragmatic context.^35,36^ Another strength is that these results are generalizable to other Canadian locations and other countries (eg, UK, US, and Australia) with similar social housing buildings. Limitations include that our cohort may have slight errors because it was based on the RPDB, which can be inaccurate due to untimely address change reporting and update lags.

## Conclusions

In this cluster randomized clinical trial, CP@clinic did not significantly affect the rate of ED visits by ambulance among participating social housing residents; however, the program did have significant effects on other aspects of resident health care utilization, decreasing system burden while directing people to appropriate primary care. Future research could determine the effectiveness of this preventive model in different populations and contexts. Health policymakers should consider CP@clinic’s impact as an upstream approach and promote widespread implementation in social housing.

## Notes

Supplement 1.
Trial Protocol

Supplement 2. **eTable 1.** Disease and Procedure Codes **eTable 2.** Medication Categories **eTable 3.** Participant Characteristics of Intervention Building Residents, Control Building Residents, and Intervention Attendees Who Had Prescription Drug Claim Records Available (≥66 Years or Eligible for the Ontario Drug Benefit) **eTable 4.** Intention-to-Treat Analysis of Medication Initiation for All Intervention and Control Building Residents **eTable 5.** Sensitivity Analysis of Healthcare Utilization and Medication Initiation Outcomes of Building Residents That Attended the Intervention Program **eTable 6.** Sensitivity Analysis of Healthcare Utilization and Medication Initiation Outcomes of All Building Residents for a Subset of Buildings (N = 26) **eTable 7.** Sensitivity Analysis of Healthcare Utilization and Medication Initiation Outcomes of Building Residents That Attended the Intervention Program for a Subset of Buildings (N = 26)

Supplement 3.
Data Sharing Statement
