# Supplementary material for: Community Paramedicine Program in Social Housing and Health Service Utilization: A Cluster Randomized Clinical Trial
Source: JAMA Netw Open. 2024 Oct 28;7(10):e2441288. doi: 10.1001/jamanetworkopen.2024.41288 (PMC11581518; doi:10.1001/jamanetworkopen.2024.41288)
Supplement: Supplement 1. — Trial Protocol [file jamanetwopen-e2441288-s001.pdf]

1.0 Introduction

1.1 Background:

Overview

Studies have shown that older adults, who comprise the population over 65 years old, account for *more than a third* of all Emergency Medical Services (EMS) calls related to cardiopulmonary conditions, diabetes and falls (1-4). Older adults with high comorbidity (>3 chronic conditions) report poorer health, take more prescription medications and have the highest rate of health care visits (5). Data from the Hamilton Central Health Links indicate that the most frequent users of EMS calls, hospital transport and hospital admissions were residents over 65 years old (6). More specifically, data from Hamilton Paramedic Service indicate that tenants in "Seniors' buildings" in underserved areas of lower socioeconomic status in Hamilton (a city in Ontario, Canada, population 520,000) are the most frequent callers to emergency 911 medical service calls. On average, there are over 200 EMS calls per day in Hamilton (J. Pyke, personal communication July 4, 2012) and with each paramedic visit and subsequent mandatory hospital emergency department (ED) physician evaluation costing around $1,044 (7,8), health care expenditures are growing out of control. Therefore, interventions that reduce the escalation of EMS calls and ED visits would be valuable to the healthcare system providing potential savings.

Recent reports highlight the need to deliver innovative, community-based care with the dual goals of enabling older adults to live safely in their own homes and alleviating related pressures on more costly care settings such as acute care hospitals and long term care services (9). Current government approaches being explored include, looking for programs which re-allocate existing resources to decrease the use of ED resources. Our team has created a multifaceted intervention (cardiovascular, diabetes, and falls risk assessment with health education/promotion and appropriate referral) in partnership with Hamilton Paramedic Service, City Housing Hamilton, and the Community Care Access Center that focuses on these particular issues experienced by older adults that often lead to EMS calls. The intervention will utilize existing city and community resources and evidence-based models. This proposal outlines the rationale for this intervention and how it will be evaluated. We believe that this research will provide evidence showing a significant difference in the number of EMS calls and ED visits between buildings with the intervention and those without it. The relative decrease in EMS calls and ED visits will have implications in terms of health care savings. We also believe that the program can improve the health behaviour of building occupants which is expected to improve health outcomes. Such findings will have significant policy implications in favour of its widespread implementation.

The Underlying Problem

Morbidity from chronic diseases and falls cause older adults to seek emergency medical care. Public Health Ontario and the Institute for Clinical Evaluative Sciences (ICES) report that chronic diseases are the leading cause of death and disability and project that they account for 89% of all deaths, *most occurring in the population over 65 years* (10). In 2007, chronic diseases (including cardiovascular diseases and diabetes) were responsible for 79% of all deaths in Ontario. These diseases diminish quality of life, resources and the potential of our communities.

Elevated blood pressure is a readily preventable cause of cardiovascular disease (CVD). The World Health Organization has identified high blood pressure as a leading risk factor for death (11). The fact that hypertension and diabetes frequently co-exist, compounds the challenges of avoiding

CVD. In Canada, 63% of adults with diagnosed hypertension also had diagnosed diabetes (12). Overall increasing incidence of diabetes forecast that 3.7 million Canadians will be living with diabetes by 2018-2019 (13).

Other than CVD and diabetes, falls contribute to morbidity among older adults. It is estimated that one in three persons over the age of 65 is likely to fall at least once each year (14, 15). The fall-related hospitalization rate for Hamilton residents has been higher than the rate for Ontario residents since 2000 and this difference appears to be widening over time (16).

CHAP model

The Community Health Assessment Program through Emergency Medical Services (CHAP-EMS) is based on the Cardiovascular Health Awareness Program (CHAP) model, a successful low cost health promotion program which targets cardiovascular risk factors, including the detection and management of hypertension. It combines individual- and population-level strategies for primary prevention and 'closes the loop' by linking participants to follow-up care. CHAP is a community-based, primary care-centered, volunteer peer-led, free of charge, CVD risk-assessment and blood pressure (BP) monitoring program combined with health education sessions for community dwelling older adults (17). In a recent community-randomized trial of **the CHAP program, intervention community seniors benefited from a significant 9% 1-year relative reduction in hospital admissions for heart failure, heart attacks and strokes** (18).

CHAP-EMS is similar to the original CHAP program in that it also closes the loop, it is located in an accessible location, enhances continuity of care, utilises blood pressure measurement with an accurate validated device, encourages referral for follow-up, cardiovascular risk assessment and education, and provides feedback of results, but aspects are modified. The modified CHAP-EMS aspects include using existing resources such as **accommodated paramedics** (medics with personal physical limitation due to injury or pregnancy) trained on the aspects of CHAP and health promotion, locating the CHAP sessions in seniors subsidized housing buildings and enhancing the cardiovascular risk screening with the diabetes risk assessment and screening for risk of falls.

Our Theory

Integrated systems have been promoted as a means to build a more affordable, effective and efficient health care system (19, 20, 21). Characteristics such as patient-centeredness, offering services across the continuum of care, strong leadership, accountability through system performance measurement, information sharing across the system, and a focus on primary care and health care teams have been consistently identified as effecting change in complex multifaceted health care systems (21). Work to improve integration therefore involves inter-related integrative processes, some fix on systems and structures, others on less tangible aspects such as teamwork (22).

A systematic review on integrated care for the elderly has highlighted the implementation of features of Wagner's model for Chronic Care (CCM) (23). The CCM is a primary–care based framework aimed at improving the care of persons with chronic illnesses. It integrates six elements (delivery system design, self-management support, decision support, clinical information services, community resources and health system organization) into a model designed to foster more productive interactions among health care providers and patients for more effective team care and improved health outcomes. (24)

This project has adapted the CCM integrated care model as a basis to explore the theorized impacts of CHAP-EMS on healthcare delivery that affect individuals and the greater system (see Figure 1). CHAP-EMS increases older adults' awareness of their own health risk factors

encourages them to manage these risk factors before any emergency event occurs, and links them with locally available community resources and services based on those risk factors. Furthermore the participants' health information (with their consent) is sent to their primary care physician so that health care services (promotion, screening, and follow-up) are coordinated to improve participants' health status. This is expected to bring better health outcomes leading to less adverse health issues, EMS calls, ED visits and hospital admissions.

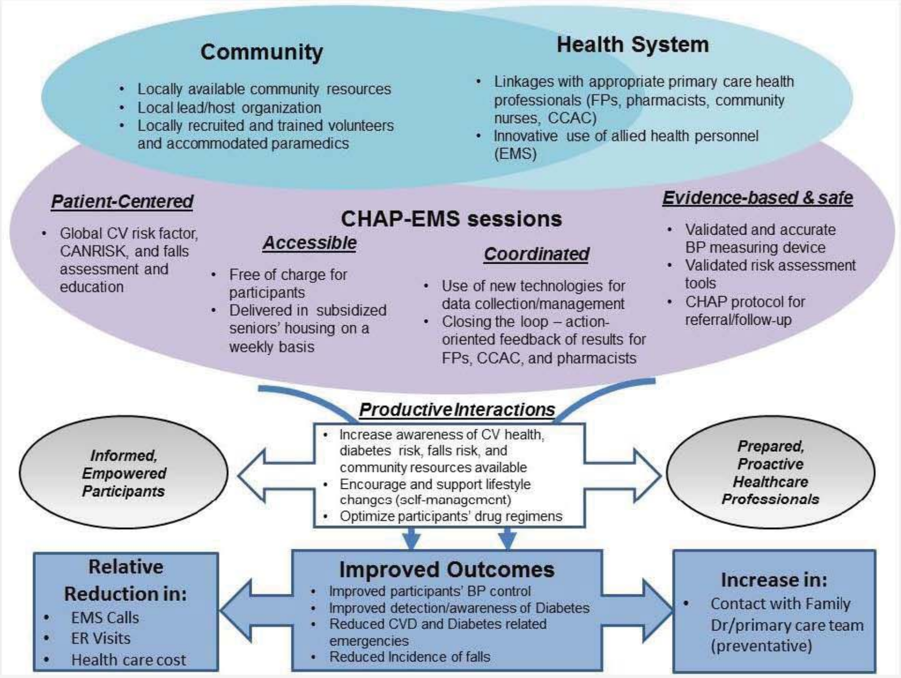

Figure 1: Theoretical Framework

Why CHAP-EMS

Treatment of hypertension can reduce the risk of stroke by up to 40%; however, many older adults are undiagnosed and untreated (25). In addition, 20% of diabetes cases remain undiagnosed (26) and diabetes is a risk factor for CVD and causes multiple organ system morbidity. To reduce the burden of CVD and diabetes, improved risk-factor screening is required (27). Previous community-based screening programs for high-risk and vulnerable populations specifically, including older adults, have been found to be both feasible and effective (18, 28, 29). Structured multi-faceted community-based falls assessments/interventions, have also been shown to reduce the risk of future falls (30, 31, 32).

The Cardiovascular Health Awareness Program's (CHAP) blood pressure and cardiovascular screening has been implemented and tested in small to mid-sized communities across Ontario (18). With our team's experience in implementing and evaluating the CHAP, we believe that expanding the program to serve **older adults living in subsidized housing, adding diabetes and falls screening,** and utilizing **accommodated paramedic personnel** to run the program will be effective

in lowering rates of EMS calls and ED visits through increasing risk factor detection and awareness, health education, and referral to appropriate services.

We are prioritizing a hard to reach population in this proposal. We are not following previous CHAP methods where the population comes out to the community program, but we are providing the community program **in the hard to reach person's home**. Older adults living in subsidized housing units report poorer health due to chronic illnesses, such as CVD and diabetes (33). Interacting psychosocial and physical factors (low income, advanced age) complicate utilization of community and healthcare services and are associated with higher risk of chronic illness and disability (34). Since older adults living in subsidized housing are defined as "senior" if they are 55 years or older, chronic diseases are occurring in younger demographics than in previous years (12-13), and individuals with low socio-economic status report poorer health (33) and increased health behaviour risk factors(10), CHAP-EMS has defined our study population as individuals 55 years and older residing in subsidized apartment buildings.

The 2012 recommendations from the Ontario Minister of Health and Long-Term Care (MOHLTC), Minister Responsible for Seniors, and consultations across the province with older adults and health care providers disclosed that often older adults cannot easily access or find a primary care provider (9). They recommend a team-based approach, including exploring the expansion of **community paramedicine** programs to support primary care access for older adults (9). Though the original CHAP program (16) was run by trained volunteers, for safety reasons, this modified program required the presence of appropriately trained health care providers. Subsidized housing buildings house individuals who are frail, seriously ill and potentially unstable and who may require immediate assistance. Paramedics are an excellent fit to deal with these emergent situations, yet also, with training, knowledgeable about health promotion and preventive medicine. A survey of community paramedicine services in Ontario in 2013(35) showed that already 6 regional services are planning to provide a chronic disease management service, and in-home lifestyle and safety evaluations. A current summary of the literature on community paramedicine (36) suggested that interprofessional collaboration is required to understand the types of services a community paramedicine program should provide. This project has already provided the collaboration between housing, paramedicine and primary care in order to develop a sustainable low cost community program.

There is much interest in other regions with this program (36) as expansion of community paramedicine is a very current topic for EMS. Other regions have accommodated staff, the same as Hamilton, and a program that utilizes them in an efficient manner is generalisable. Though Public Health is supportive of this program, they are currently not able to take on accountability for additional staffing (public health nurses to perform the assessments), since the mandate of Public Health is to improve population health and not individuals' health. The use of accommodated paramedics allows for highly skilled and trained individuals to be reallocated for a worthwhile community healthcare activity, while still receiving active pay for their service. Paramedic training prepares these individuals to accurately assess the patient's health status and the environmental context, and connect patients with primary care physicians, visiting nurses, and community services including falls prevention (37-41); these skills can be expanded to provide non-urgent health care services care in areas of community need. The **'accommodated' paramedics** utilized in this program are unable to assume traditional paramedic duties due to personal physical limitations such as pregnancy or injuries. These limitations, though rendering traditional paramedic duties unsuitable, still allow for health promotion work. Accommodated paramedics remain in the employment of the

paramedic service for the duration of their condition, but are often not using their skills and training in the community. Utilizing them for work they are able to do, will be much more cost-effective and feasible than the placement of a family physician or other health practitioners, who would need to be funded from other external and potentially unavailable sources, compared to accommodated paramedics who have the skills and are **already funded**. Personal conversation with EMS administrators from multiple regional agencies indicate that there are two to upwards of 15 paramedic staff on accommodated duty on any one day shift and that there is often not enough work considered as light duties for all these individuals. In this project paramedics will be accountable to their respective paramedic service for their actions, who act as an extension of the MOHLTC and the Ambulance Act of Ontario (personal communication with Chief Sanderson, Hamilton EMS).

In October 2011, Hamilton Paramedic Service, collaborating with the Community Care Access Center (CCAC), implemented the Community Referral through EMS (CREMS) program to increase the value of EMS services by using paramedicine professionals in new ways, improving the access of vulnerable older adults to preventive health care. The implementation of CREMS shows the willingness of Hamilton paramedics to accept expanded roles and work with community agencies, although the CREMS and CHAP-EMS programs are different. The CREMS program is based on referrals to community services occurring after EMS has been called to the residence and is only acted upon for that residence. In contrast, CHAP-EMS is a prevention and education program that provides regularly scheduled sessions within the apartment buildings (i.e. is not dependent on an initial EMS call) to **all residents of that building**. While CREMS provides referrals to CCAC, CHAP-EMS provides these referrals when needed but also aims to reduce EMS calls and hospitalization through monitoring and reducing lifestyle risk factors (e.g. high blood pressure and prediabetes) and referring to other community programs (e.g. local wellness programs).

Our pilot study results (Section 2.14) show that CHAP-EMS has the potential to deliver quality care and reduce the burden of chronic disease among the elderly living in subsidized housing eventually leading to a reduction in EMS calls and ED visits. However, CHAP-EMS needs to be formally evaluated.

1.2 Objectives and research questions: Our primary objective is to evaluate the CHAP-EMS program in order to answer the following research questions:

- Will there be a **difference in the rate of EMS calls** in subsidized older adults' housing buildings receiving the CHAP-EMS programs compared to buildings not receiving the program?
- Will there be a **change in measured systolic and diastolic blood pressure** in older adult residents living in subsidized older adults' housing after receiving the CHAP-EMS program compared to their own baseline measurement?
- Will there be a **difference in 10-year diabetes risk, as measured using the CANRISK tool,** in older adult residents living in subsidized older adults' housing after receiving the CHAP-EMS program (healthy lifestyle education) compared to their own baseline measurement?
- Will there be a **difference in the health perceptions, behaviours, intentions regarding behaviours, health literacy, and knowledge of resources** in older adult residents living in subsidized older adults' housing after receiving the CHAP-EMS program compared to their own baseline measurement and compared to older adults in a building not receiving the

program?

- Will there be a **difference in health seeking behaviour (e.g. number of hospital emergency room visits and primary care visits)** by older adult residents living in subsidized older adults' housing compared to baseline and compared to older adults in a building not receiving the program?

Our secondary objectives are to assess the program and process of implementing CHAP-EMS in terms of number of participants identified with high blood pressure; number of participants at high risk for current or future diabetes and falls; number of referrals to the CCAC; number of participants who have made lifestyle modifications; number attending community wellness and physical activity programs; and process evaluation (e.g. completion of risk assessments).

1.3 Significance of the study: Recent Institute of Clinical Evaluative Sciences(ICES) reports identify that the amount of health care services used by older adults is driven mainly by the number of chronic conditions they have (42, 43). Consequently, ICES recommends that health care providers need to work actively with older adults to prevent new chronic conditions and manage existing conditions to avoid complications (42). Furthermore, one of the key recommendations of the Ontario Ministry of Long Term Care report is the development and expansion of the role of allied health professionals to provide the right care in the right place at the right time to vulnerable community living older adults, and in particular "*to explore the development and expansion of* ***community paramedicine***." (9)

This study addresses the prevention aspects for older adults with an innovative expansion of the paramedic role, in collaboration with primary health care providers, to increase the reach of healthcare and access to vulnerable older adults. The goals of this project will address cost efficiencies through fewer calls to EMS, fewer visits to the ED, and increased access to community support systems through follow-up linkages and visits with primary care providers and CCAC. The intervention re-allocates and utilizes resources which are already available. All of these positive changes are expected to be beneficial to the health care system. Although there may be an increase in other primary care visits (family medicine visits and walk-in clinic visits) as a result, this usage should be appropriate and beneficial to the overall improvement of the health of older adults. If the study results support that the CHAP-EMS program is beneficial in reducing ED use and health risks, this information will help inform decision makers in policy planning for expanding the paramedic role in other regions. Furthermore, the study data will provide the required information for future study, including both a scale-up and for a larger multi-centre, multi-community study to determine effectiveness.

1.4 Review of literature: Cochrane, Medline, EMBASE, Global Health, and Psyche Info databases were searched to identify any previous studies that examined the relationship between the use of paramedicine and improved EMS call statistics, health outcomes or other outcomes. There were no systematic reviews, but primary studies were found related to role expansion for paramedics.

Studies regarding the expanded role of paramedics relating to health promotion were limited to case studies and were mostly in rural areas in Australia (44-46). In many cases, paramedics were seen as an underused human resource in rural and remote areas (44). Expanded roles included both clinical and primary care such as community education and engagement, preventive services, treatment of minor illness (and locally endemic conditions), and promotion of lifestyle change to

prevent and manage chronic disease (44, 46). A qualitative evaluation showed that these roles were acceptable to the paramedics and were appreciated by the community. Though CHAP-EMS will incorporate similar health promotion activities, we will be implementing our intervention in a densely populated urban setting therefore limiting the length of time available for each participant session.

Another study in the United Kingdom evaluated expanding the roles of paramedics in responding to calls from the elderly living in urban areas (47). Paramedics were given the additional role of referring patients to a general practitioner, district nurse, and/or community social services when needed.

The expanded overall role of paramedics, including health promotion and referral to appropriate health service providers, has been successfully implemented in other settings such as in Australia and the UK. The CHAP model of assessment of older adults in the community for risk of hypertension has also been proven to be an effective intervention resulting in fewer cardiovascular-related hospital admissions (18). As well, CHAP was successfully expanded to include a diabetes risk assessment component in the Community Health Awareness of Diabetes (CHAD) program (48).

2.0 Research Methods

2.1 Study Design: The intervention will be implemented in four to six communities. Depending on the size of the community, four to ten subsidized seniors' buildings managed by local housing organizations will be paired based on their characteristics and randomly allocated to receive or not receive the CHAP-EMS program. The program will be delivered for 1 year in the intervention buildings. Outcomes will be analyzed using parallel comparisons (of intervention versus control buildings) as well as comparisons before and after the intervention is implemented. Process evaluation will be done after the 1-year study implementation and will assess the program implementation in terms of efficiency of the processes in its delivery, participation rates, compliance, and ways of improving it.

Year (Research Phase)	Months	Group (4 buildings in each group)	
		**Intervention**	**Control**
Year 1 (Pre-intervention)	1-9	Planning, networking and capacity building, training and preparation	
	10-12	Survey	Survey
Year 2 (Intervention)	1-12	CHAP-EMS Implementation	Usual Care
Year 3 (Post-intervention)	1-3	Survey	Survey
	4-12	Collecting administrative data, accessing primary care physicians and chart reviews, data analysis, data dissemination, knowledge translation	

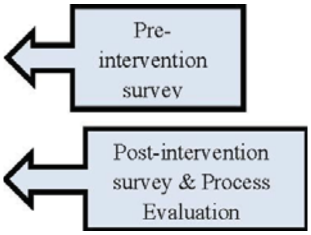

Figure 2: Research Design

2.2 Inclusion/exclusion criteria: Subsidized seniors' buildings managed by Community Housing will form the sampling frame. Buildings will be selected based on the following criteria: 60% or more of the apartment units are occupied by residents 55 years or older; there should be at least 100 apartment units.

All residents 55 years and older of the selected buildings will be included as potential participants for the individual level outcomes. Transients (staying in the buildings for less than 3 months) will be excluded from the outcome analysis. However, the intervention will be offered to all residents of the selected buildings whether or not they end up as participants in the outcome analysis. Participation in CHAP-EMS program will be voluntary. The program will be advertised to the target population using a combination of presentations to tenant associations, word of mouth, and posters.

2.3 Allocation of intervention: The subsidized seniors' buildings included in this study will be medium- to large-sized apartment buildings with a high proportion of aging residents (defined as >60% over the age of 55 years old). The buildings will be paired based on geographic location, numbers of housing units within the building, proportion of units occupied by older adults, the known existing rate of EMS calls received by the local paramedic service (number of calls/100 apartment units) in the two years prior to the intervention period. For each pair, one building will be randomized to receive the intervention and the other will not receive the intervention (control group). Block randomization will be carried out using computer generated random allocation.

2.4 Sample size: Given that our main outcome will be rates of calls per 100 residents per year; we calculated our sample size using the formula for the Poisson test. The average rate of calls for our pilot sample was 44.06 EMS calls per year per 100 residents older than 65 years (information supplied by J. Pyke, Hamilton Paramedic Service). If we assume a difference of 10% (a difference of 4.4 EMS calls per 100 residents per year) between the intervention and control groups, with 0.8 beta and alpha of 0.05, we would need a sample of 131 per group. This is a conservative estimated effect size since the pilot study showed a 32% reduction in EMS calls (see Section 2.14). Also, a difference of 10% was considered operationally significant by Hamilton Paramedic Services (Personal communication with B. Mcleod); this would translate to a cost avoidance of $8,000 per month per building based on an average building size of 200 residents.

The sample size will be a minimum of 700 participants per group and a minimum of 8 buildings across all sites. Considering the Design Effect (cluster sampling by buildings), we can accommodate an Intracluster correlation coefficient (ICC) of up to 2.5% given our proposed sample size. There is no literature regarding an ICC for a similar study.

The targeted sample size of residents completing the Health Awareness and Behaviour Tool (HABiT) is 228, both pre- and post-intervention, for a minimum of 456 surveys. This is based on an expected difference of 15% between the intervention and control buildings and an ICC of 0.1. The number of residents sampled from each building will be proportionate to the number of units in the building.

2.5 Recruitment process: Recruitment will be organised in a multi-faceted way. Existing building-based wellness groups and Tenants' Associations will identify leaders from among the residents of the buildings who can champion the CHAP-EMS program. These leaders will be asked to participate in information sessions for building residents and to spread their knowledge about the program through the building in general. The program will also be advertised to the target population using posters in the intervention buildings. Posters will be translated into other languages as needed by the population composition in the buildings chosen. The pilot study showed this method of recruitment

was feasible and that participation rates were acceptable. Pilot data shows that after 8 months offering a one day week intervention, there were approximately 55 participants (from a total number of 234 senior residents; a participation rate of 23.5%). Our pilot data shows that this rate will increase with word of mouth amongst residents as time progresses; there are around 3 to 8 new participants attending each month with 48 (87% of those attending the program) having at least 2 visits. Based on this, we estimate that 30-40% of residents will come into contact with the program during the 12 month intervention.

Participants in the control buildings will receive usual care and will not be recruited for any intervention. The only contact the researchers will have with the participants of the control buildings will be to obtain survey responses regarding their health perceptions and behaviours. Consent for participation in the survey will be incorporated in the survey package (detailed in Section 2.9: Data gathering procedures).

2.6 Research Intervention: The intervention buildings will receive the CHAP-EMS program; main elements include BP, diabetes and falls risk assessments, health education/promotion, targeted referral to appropriate in-house wellness sessions and community resources (e.g. CCAC), as well as, identification and referral of high risk patients, and referral of participants' health information to their regular physician. CHAP-EMS will be held weekly at the intervention sites by paramedics from the local paramedic service. The Paramedics delivering CHAP-EMS will have undergone a structured training program (3-4 hours of online, interactive training modules, including case studies, and the observation of a CHAP-EMS session led by another paramedic) to assure intervention fidelity. The training program was developed by a family physician (GA), public health nurse (BM), and paramedic (BM) (all co-investigators).

Individual participants (residents of the seniors' buildings) voluntarily attend and will complete the informed consent process with a paramedic on their first visit. Following consent, they will have their risk factors assessed and entered into a database, and are advised to make lifestyle changes (if applicable) with the help of available community resources. The CHAP-EMS intervention has been adapted from over 12 years of experience from the development and evaluation of the Cardiovascular Health Awareness Program (CHAP) in various communities across Ontario and Alberta (www.chapprogram.ca). However, CHAP-EMS is a *different model* as it focuses on an urban, in-home, low socio economic status, frail elderly population who are known high EMS-call users. This is expected to have a bigger impact because it includes diabetes, falls, lifestyle, and collaboration with new partners not previously part of CHAP (CCAC, Hamilton Paramedic Services, and Community Housing). The assessments in CHAP-EMS include blood pressure and cardiovascular risk screening, *but also* diabetes risk-assessment using the validated CANRISK tool (49), environmental risk of falls, and the "Timed up and go" (TUG) test (50, 51). Participants with a moderate-to-high score on CANRISK will be asked to return for a fasting blood glucose capillary test, where the paramedic will guide the participant through pricking his or her own finger with a lancet and applying a drop of blood to a test strip to measure fasting blood sugar. The assessments will be collected on the CHAP-EMS database (a computer-based questionnaire which collects participant information, then calculates risk factors and summarizes them for targeted interventions such as referrals).

The CHAP-EMS database uses a pre-specified algorithm developed to direct participants to the appropriate services. Participants identified as high risk will immediately be referred to appropriate health care resources. Based on a moderate risk profile and a needs assessment, participants will be

referred to community resources to assist them in managing their health. These community resources are run by Community Housing and Public Health and include age-appropriate physical activities, speakers on healthy eating, social engagement opportunities with cooking demonstrations and referrals to local community resources as required (e.g. EatSmart, community food advisors, local recreation department, Medscheck program and CCAC for falls prevention education). Follow-up for identified concerns will be provided through linkages with primary health care providers and CCAC community referrals. Geographical information system (GIS) mapping will provide a visual display of local availability of health care resources and locations where older adults can be safely active and obtain healthy food.

All participants will be encouraged to attend CHAP-EMS program sessions regularly for BP monitoring since even participants with normal or controlled BP can experience fluctuations due to medications /compliance issues, co-morbidities and personal events. Furthermore, CHAP-EMS also provides social support, education, and encouragement to pursue lifestyle changes. CANRISK assessments will be repeated at 6 month intervals to assess change. **Consenting participants will have their BP and risk profile sent by fax from the CHAP-EMS database, to their regular physicians** (and those without will be referred to CCAC for assistance with locating suitable local Family Physicians). CHAP-EMS will be cost-free for all building residents but will target those aged 65 years and older. Since the intervention was pilot tested in a seniors' building already, this building will not be included in the study. The program will be advertised using a combination of approaches discussed above including presentations and posters.

In the control buildings, residents will receive usual care through their family physician (for those who have one) or their regular primary care services and services provided by Community Housing.

During the CHAP-EMS sessions, where significant mental, emotional or physical health issues are identified, the paramedic will refer to the appropriate service (i.e. EMS for urgent health concerns, CCAC, family physician, etc.)

2.7 Duration of intervention and frequency of follow up: CHAP-EMS will be implemented in the intervention buildings for **12 months**. There will be 2 trial evaluation periods (pre-intervention/post-intervention) 12 months apart. Process evaluation will be done at the end of the 12-months intervention.

2.8 Primary and secondary outcome measures: The **primary outcome** will be the **rate of EMS calls** (# of calls for building by residents aged 55 years or more/ # of residents aged 55 years or more in the subsidized seniors' building). The number of calls for each building will be available from the EMS administrative database. The **secondary outcomes** will be change in measured systolic and diastolic blood pressure ; difference in 10-year diabetes risk (CANRISK); difference in health perceptions, behaviours, intentions regarding behaviours, health literacy, and knowledge of resources (HABiT survey); and difference in health seeking behaviour (e.g. # of ED visits/population 55 years and older) based on administrative databases. See Table 1 for details on the measures and data sources being used.

Process evaluation measures will include participation rates (number of participants attending – initial attendance and repeat visits), program delivery (e.g. completion of risk assessments), and other program evaluation measures (e.g. detection rates for hypertension and diabetes).

An **economic analysis** will also be performed, looking at the cost impact and cost effectiveness of the program of alterations in ED visits, admissions and primary care visits in the short and long term.

2.9 Data gathering procedures: Rates of EMS calls, ED visits, primary care visits, and other health care utilization will be collected from the **administrative database of the local paramedic services, hospital ED databases, Institute for Clinical Evaluative Sciences databases, the CHAP-EMS database and consenting participants' primary care charts**. See Table 1, below, for details. Administrative data will be collected pre- and post-intervention, and retrospectively for the 12 months of intervention. These outcomes will be analyzed at the cluster level.

Health perceptions, behaviours, intentions regarding behaviours, health literacy and knowledge of resources, related to chronic disease risk factors will be collected from consenting participants using an **interviewer-led survey** called the Health Awareness and Behaviour Tool (HABiT), loosely based on knowledge and behaviour surveys of the Canadian Hypertension Education Program (CHEP) and the CHAD Program (52). The questionnaire will have been pretested and validated in a sample of older adults in Ontario. Each item will be assigned a score and participant questionnaire results will be summarized as numerical scores. These outcomes will be analyzed at the individual level and linked to administrative databases.

There will be 2 periods of data collection with the HABiT survey; a pre-intervention survey for residents of the intervention and control buildings administered up to 3 months prior to the intervention and a post-intervention survey administered up to 3 months immediately post intervention. Residents will be notified about the surveys through presentations at tenant engagement meetings, word-of-mouth, and posters within the buildings. Surveys will be administered by trained college/university students and paramedics to a random sample of residents in the building. Randomization will occur by visiting every n^{th} apartment (e.g. every 5th), depending on the building size. If more respondents are needed, the non-sampled apartments would then be randomized again, and this would continue until the target sample size for that building is achieved. If more than one resident is in the unit and willing to participate, the individual with either the most recent or the closest birthday will be selected according to the randomization protocol.

Physical measures (i.e. blood pressure, weight), risk assessment scores, and process outcomes will be collected through the CHAP-EMS electronic database, which will be used during the intervention.

Table 1: Summary of Objectives, Data source, and Analysis

Objective/Outcome	Data Source	Data Analysis
Decrease in rate of EMS calls (Primary Outcome)<br>Level of analysis: Building<br><br>Measure: Number of calls by residents aged 55 years and older per 100 residents aged 55 years and older in each building	EMS administrative data<br>National Ambulatory Care Reporting System (NACRS)*	• Longitudinal change before / during the intervention<br>• Multi-level modeling of factors affecting outcome<br>• Pairwise comparison between intervention / control group during 12 month intervention
Decrease in ED visits (Secondary	Hospital/ED database	

Outcome)<br>Level of Analysis: Individual and Building<br><br>Measure: Total number of ED visits for each building, 12 months pre-intervention and 12 months during intervention<br>Measure: Total number of ED visits by CHAP-EMS participants in 12 months pre- invention and 12 months during intervention	EMS administrative data<br>ERCLAIMS database (ICES derived)*<br>National Ambulatory Care Reporting System (NACRS)*	
Increase in primary care visits (Secondary Outcome)<br>Level of Analysis: Individual and Building<br><br>Measure: Total number of primary care visits for each building, 12 months pre-intervention and 12 months during intervention<br>Measure: Total number of primary care visits by CHAP-EMS participants in 12 months pre- invention and 12 months during intervention	CHAP-EMS database (self-report)<br>EMR Chart audit<br>OHIP Claims History Database*	
Increase in other health care utilization (Secondary Outcome)<br>Level of Analysis: Individual and Building<br><br>Measure: For each form of health care utilization below, total number of interactions for each building, 12 months pre-intervention and 12 months during intervention<br>Measure: For each form of health care utilization below, total number of interactions by CHAP-EMS participants in 12 months pre- invention and 12 months during intervention<br><br>Health care utilization:<br>- Any healthcare contact (quarterly)<br>- Non-GP Specialist visits	CHAP-EMS database (self--report)<br>CONTACT database (ICES derived)*<br>OHIP Claims History Database*<br>Discharge Abstract Database (CIHI)*<br>Home Care Database (OACCAC)*	

- Physician claims, other than primary physician<br>- Hospital visits<br>- CCAC:<br>- Public Health Centre/ Community Care Centre, Nurse visit<br>- Public Health Centre/ Community Care Centre, Allied Health visit (e.g. physiotherapy, nutritionist)<br>- In-Home Nurse visit<br>- In-Home Allied Health visit (e.g. physiotherapist, nutritionist)		
Improved perceptions and behaviours regarding chronic disease (Secondary Outcome)<br>Level of Analysis: Building<br><br>Measure: Scores for health-related quality of life, health literacy, health behaviour, risk awareness, self-efficacy, and intention to change, pre- and post-intervention	Interviewer-led questionnaire	• Pairwise comparison between intervention / control group before / after intervention
Increased HTN and T2DM medication use among residents aged 66 years and older (Secondary Outcome)<br>Level of Analysis: Individual and Building<br><br>Measure: HTN and T2DM medication use among residents aged 66 years and older, for each building, 12 months pre-intervention and 12 months during intervention<br>Measure: HTN and T2DM medication by CHAP-EMS participants aged 66 years and older in 12 months pre-invention and 12 months during intervention	Ontario Drug Benefit (ODB) Program Database*<br>EMR Chart audit	• Longitudinal change before /during intervention<br>• Pairwise comparison between building-level data for intervention / control group and before / after
Number of referrals to CCAC, Community Housing, primary care	CHAP-EMS database<br>EMR Chart audit	• Descriptive analysis

doctor, specialists, allied health professionals (Secondary Outcome)<br>Level of Analysis: Building<br><br>Measure: Number of referrals to each service/health care professional (see below), 12 months pre-intervention and 12 months during the program.<br><br>Professional/Service:<br>- Primary Care<br>- Specialist<br>- Other health professionals<br>- CCAC<br>- Community Housing<br>- Wellness Program<br>- Other community programs	EMS administrative data<br><br>OHIP Claims History Database*<br>Home Care Database (OACCAC)*	
CHAP-EMS detection rate new HTN and T2DM; poorly controlled HTN (Secondary Outcome)<br>Level of Analysis: Building (Intervention Only)<br><br>Measures: Number of new HTN, poorly controlled HTN, and T2DM cases in building residents and number detected through CHAP-EMS	CHAP-EMS database<br>EMR Chart audit<br>Ontario Hypertension Database (ICES derived)*<br>Ontario Diabetes Database (ICES derived)*	• Descriptive analysis
CHAP-EMS participation rates (Process measure)<br>Level of Analysis: Building (Intervention Only)<br><br>Measures: Number of CHAP-EMS participants over the number of eligible building residents	CHAP-EMS database	
Process outcomes (e.g. risk assessment completion) (Intervention buildings only)<br>Level of Analysis: Building (Intervention Only)<br><br>Measures: Program implementation, assessment completion, fidelity, etc.	CHAP-EMS database	

*Housed at the Institute for Clinical Evaluative Sciences (ICES)

2.10 Data Analysis: We will use an **intention-to-treat analysis** and **generalized estimating equations** (GEE) assuming a Poisson distribution and an exchangeable correlation structure within a building to evaluate health care utilization, blood pressure, and risk assessment scores, as well as to identify factors contributing to these outcomes. Rate of EMS calls will be analyzed at the building-level only, using GEE. We will perform **pairwise comparisons** of outcomes for each month during the pre-intervention and intervention periods. We will identify trends in the outcomes during the pre- and post-intervention observation periods. Subgroup analysis by clusters (each older adult building) and by location will be performed. The HABiT survey data will be summarized as a numerical score representing the participants' perceptions and behaviours regarding CVD and diabetes, and analysed with GEEs. The intra-cluster correlation coefficient (ICC) will be computed to determine the effect of clustering (by the building participants reside in) on the participants' scores. Other outcomes will be analyzed using descriptive statistics.

All analyses will be performed using SAS 9.2 (Cary, NC). All analyses requiring health administrative data will be performed using linked, deidentified records at the Institute for Clinical Evaluative Sciences (ICES), a Prescribed Entity under Section 45 of Ontario's Personal Health Information Protection Act. Statistical significance will be set at alpha = 0.05 and adjusted using the Bonferroni method for secondary analyses. Results will be reported as rate ratios, 95% confidence intervals and associated p-values.

Two monthly review and analysis of CHAP-EMS data will occur to assess for errors in data entry and assure fidelity to the planned intervention (monitor referral patterns by EMS personnel and proper use of the screening tools). The main outcomes (rates of EMS calls, visits to the ED, and primary care visits) will be analyzed at the beginning of the intervention (pre-intervention data) and after the 12 months intervention period (post-intervention data and full data analysis).

Both CHAP-EMS intervention and health care resource utilization/costs will be collected for the economic analysis, with a focus on whether the up-front cost of the CHAP-EMS program is offset by other health care cost savings. Decision analytic modelling techniques will be used to project final outcomes like life years and quality adjusted life years from intermediate outcomes measured during the evaluation period. Our economics team has extensive experience in conducting trial-based and modelling-based evaluations aligning with the needs of health policy makers.

2.11 Issues regarding compliance: The program is voluntary; one outcome is residents' attendance. Pilot data showed that of 56 participants who had attended since program inception, 70% returned for repeat visits; indicating a low likelihood of attendance issues. For the survey, residents will be given $10 gift certificates on survey participation and we will continue to follow the random sampling strategy until we meet the target sample. Residents who are not part of the random sample can also complete the survey to receive the gift card. We will utilize the expertise of an advisory committee (See section 3.1 of Trial Management) to maximize strategies to improve compliance. We will emphasize to all participants that the survey will be anonymous and no identifiers will be requested to ensure privacy.

2.12 Control of bias: For the primary outcomes, we will collect at least one year of data before the intervention starts and also use a parallel design (for pairwise analysis) to examine possible effect of seasonality. For the 'perceptions and behaviour survey', the nature of the intervention prevents blinding. Participants will not be informed about the group comparisons. Surveys will be anonymous to decrease the possibility of social desirability bias. We will emphasize that the survey is

anonymous and no identifiers will be requested to ensure privacy. Audio recordings of the FGD and KII will be used to ensure accuracy. Individual coders will analyze qualitative data and compare coding to ensure triangulation of the data. A journal of study activities will be kept so that procedures are well documented and can be replicated. The questionnaires used will also be pre-tested for clarity.

2.13 Loss to follow-up: We do not expect a loss to follow-up (due to death or transfer of residence) of more than 5% since this is a 1 year intervention study. Yearly death rates for males and females are 5.85 and 5.59 per thousand (54), unlikely to affect our study, despite possible slightly higher rates due to the socio-economic status of the population. We do not anticipate large fluctuations in residents occurring due to multiple evictions or transfers. Information from City Housing show an estimated eviction and transfer rate of between 5-7% in similar buildings (personal communication from Amy Rooke, City of Hamilton Housing). This event will be monitored through our housing partners.

2.14 Pilot study results: Our pilot study of the CHAP-EMS program (offered one day a week for **8 months**) in one older adult housing building, we have a total of over **580 participant visits** by **56** residents (3-5 new participants a month). Over **50% of participants had elevated blood pressure** readings and more than **48% also had a high risk for developing future diabetes** based on their CANRISK scores. Among those who had elevated blood pressure, 80% were previously diagnosed with hypertension and were taking medications. Medication changes were made by their Family Physician. The others, who were not previously diagnosed, were referred to their regular physicians and some subsequently diagnosed with hypertension. **Prevalent risk factors** among the participants were high waist circumference (68.5%), elevated Body Mass Index (63%), low physical activity (40.7%), smoking (33.3%), high salt intake (33.3%), high fatty food intake (33.3%), and low fruit and vegetable intake (29.6%). Participants who attended CHAP-EMS also connected with available community resources to assist them in managing their health. Preliminary reports from Hamilton Paramedic Service calls indicated that there was a **32% reduction in the number of calls** from the pilot site compared to the previous year.

3.0 Study management and utilization of results

3.1 Day to day management: Data from each participant of the CHAP-EMS sessions will be collected, coded and entered into the electronic database on a password-protected computer by paramedic staff. A research coordinator will regularly collect and analyze this data and store it in an encrypted database. All questionnaires, qualitative transcripts and confidential participant data, and consent forms, will be kept secured and private in a locked filing cabinet. Data will be managed by the research team.

3.2 Principal applicant and co-applicants: The partners for the current study are the Department of **Family Medicine** of McMaster University, **Public Health** Services, City of Hamilton, **Heart and Stroke Foundation of Ontario**, **Canadian Diabetes Association**, **Community Care Access Centre**, **City Housing Hamilton** and the **Local Health Integration Network** 4.

Dr Gina Agarwal MBBS PhD MRCGP CCFP FCFP, an Associate Professor in the Department of Family Medicine, both a clinician and a researcher and she will serve as the PI for the study. She will head the entire research team and manage overall issues regarding the research and intervention.

Dr Lisa Dolovich PharmD, MSc is the Research Director, pharmacist, and an Associate Professor in the Department of Family Medicine at McMaster and co-leader of the CHAP program of research. She will provide mentorship around project leadership, methodological and implementation expertise to the team. She has been involved with CHAP since 2000.

Beatrice McDonough BScN, MScN, MSc, is a public health nurse with Public Health Services at the City of Hamilton and a Faculty of Nursing and the Department of Family Medicine at McMaster University will facilitate the advisory collaborative committee and promote activities to strengthen partnerships. She has been involved with CHAP since 2001.

Brent McLeod BA, MHM, MPH, AEMCA, ACP, is a Paramedic Supervisor with the Hamilton Paramedic Service and will assist in developing the research methodology, developing training modules for the paramedics, and supervising the paramedics administering the CHAP-EMS.

Drs Janusz Kaczorowski and *Larry Chambers* are co-leaders of CHAP program. *Dr Kaczorowski* is a Professor and the Research Director, Department of Family Medicine, Université de Montréal, medical sociologist, and member of the leadership team in CHEP, Hypertension Canada and the Canadian Stroke Strategy. *Drs Kaczorowski and Chambers* will provide methodological support and guidance. They have been involved with CHAP since 2000.

Dr Lehana Thabane and *Ron Goeree* are Professors of the Clinical Epidemiology & Biostatistics, McMaster University. *Dr Thabane* will provide senior statistical guidance and advice on the methodology employed and statistical analysis of the trial. *Ron Goeree* will provide leadership and guidance around the economic analysis outcomes of the trial.

Michael Paterson is a Scientist with ICES and an Assistant Professor in the Department of Family Medicine at McMaster University. He will provide expertise in linking the CHAP-EMS data to the appropriate administrative databases housed by ICES and will oversee the data linkage and analyses conducted at ICES.

3.3 Advisory committee: The advisory committee (paramedics, CCAC, Public Health, Community Housing, and the research coordinator) will be responsible for providing on the ground logistical support, problem-solving for the project and reviewing study results. They are not investigators but are collaborators serving on the research team and have provided letters of collaboration. The team approach provides an opportunity for knowledge translation and enhances shared communication across the partner organizations. There will be no formal data safety monitoring board however the advisory committee will review and provide advice regarding any incidents that have occurred.

3.4 Health service research issues: This study is in keeping with the Living Longer, Living Well strategy (9) for utilizing allied health professionals more appropriately and extending their roles within health promotion and disease prevention. It addresses the question of the effectiveness of a novel form of health services delivery on health care processes, health outcomes, and health resource utilization.

3.5 Knowledge translation and implications: The **trainee graduate student** will be encouraged to adopt and expand the evaluation of secondary research questions based on interest; or to devise a question on their own related to the project's direction. The project will form the basis of a thesis.

Involving the advisory committee of knowledge users (Hamilton Paramedic Services, Community Care Access Centre, Public Health, City Housing Hamilton) in a knowledge translation and exchange process will result in opportunities to use the results **when making**

decisions about future health programs, policies and /or practices. The committee will be involved with the collecting and analyzing of the data, developing recommendations for Hamilton City Council and the dissemination of results. Dissemination includes development of an online training tool for paramedics regarding their expanded role into the health promotion/non-urgent aspect of preventive health care. The **educational tool** developed by our team will utilize the format and platform used for paramedic education in Ontario (CPER). Paramedicine decision-makers can share the new tool and the research results with their provincial and national EMS bodies to promote this new knowledge and expand the CHAP-EMS program.

City Housing and Public Health will share our results and tailor their messages accordingly to enhance sustainability actions. **Potential audiences** could be decision makers, policy makers, funding bodies or knowledge brokers. **Sustainability actions** will involve working with relevant groups such as health care professionals, service providers, and community health support organizations focusing on older adults (e.g. LHIN Community Collaborative) and decision makers from local municipal and provincial governments. Evaluation of the impact of these approaches will be captured though qualitative process evaluation (KII interviews described above). Results will be presented in conferences and published in peer-reviewed and open access journals to reach broad audiences to enhance research uptake.

Reference List

1. Platts-Mills, T. F., Leacock, B., Caba, J. G., Shofer, F. S., & Mclean, S. A. (2010). Emergency medical services use by the elderly: Analysis of a statewide database. *Prehospital Emergency Care, 14,* 329-333.
2. Svenson, J. E. (2000). Patterns of Use of Emergency Medical Transport�: A Population-Based Study. *American Journal of Emergency Medicine, 18*(2), 130–134.
3. Weiss, S.J., Earnst, A. A., Miller, P., Russel, S. (2002). Repeat EMS Transports among elderly emergency department patients. *Prehospital Emergency Care,6*, 6-10.
4. Wofford, J. L., Moran, W. P., Heuser, M. D., Schwartz, E., Velez, R., & Mittelmark, I. M. B. (1995). Emergency Medical Transport of the Elderly�: A Population-Based Study. *American Journal of Emergency Medicine, 13*(3), 297-300.
5. Canadian Institute for Health Information (2011). *Seniors and the Health Care System: What is the impact of multiple chronic conditions?* Accessed from: https://secure.cihi.ca/free_products/airchronic_disease_aib_en.pdf
6. Hamilton Central Health Link (2013). *Hamilton Central Health Link Business Plan.* Accessed from: http://www.hamilton.ca/NR/rdonlyres/A1C7A6DC-7041-4964-AD1E-B374E2CB33AC/0/Apr2281BOH13016__Hamilton_Central_Health_Link.pdf
7. Longwoods.com Healthcare Quarterly (2009). *CIHI Survey: ED Spending in Canada: A Focus on the Cost of Patients Waiting for Access to an In-Patient Bed in Ontario*. Accessed from: http://www.longwoods.com/content/20411.
8. TheSpec.com (2010). *Code red: Band-Aid fixes getting us nowhere.* Accessed from: http://www.thespec.com/news/article/21318--code-red-band-aid-fixes-getting-us-nowhere .
9. Sinha, S. K. (2012). *Living Longer, Living Well: Highlights and Key Recommendations*. Accessed from: http://www.health.gov.on.ca/en/common/ministry/publications/reports/seniors_strategy/docs/seniors_strategy.pdf
10. Manuel, D.G., Perez, R., Benett, C., Rosella, L., Taljaard, M., Roberts M., et al., *Seven more years: The impact of smoking, alcohol, diet, physical activity, and stress on the Health and life expectancy of Ontarians*. Accessed from: http://www.oahpp.ca/resources/documents/reports/seven_more_years/PHO-ICES_SevenMoreYears_Report_web.pdf
11. World Health Organization (2009). *Global Health Risks*. Accessed from: http://www.who.int/healthinfo/global_burden_disease/GlobalHealthRisks_report_full.pdf
12. Public Health Agency of Canada (2010). Report from the Canadian Chronic Disease Surveillance System: Hypertension in Canada, 2010. Accessed from: http://www.phac-aspc.gc.ca/cd-mc/cvdmcv/ccdss-snsmc-2010/pdf/CCDSS_HTN_Report_FINAL_EN_20100513.pdf
13. Public Health Agency of Canada (2011). *Diabetes in Canada: Facts and figures from a public health perspective*. Accessed from: http://www.phac-aspc.gc.ca/cd-mc/publications/diabetesdiabete/facts-figures-faits-chiffres-2011/highlights-saillants-eng.php
14. World Health Organization (2007). *WHO Global report on falls prevention in older age*. Accessed from: http://www.who.int/ageing/publications/Falls_prevention7March.pdf.
15. Scott, V. J., Peck, S., Kendall, P. (2004). *Prevention of falls among the elderly*. Accessed from: http://www.health.gov.bc.ca/library/publications/year/2004/falls.pdf
16. Hamilton Public Health Services (2011). *Falls related injuries*. Accessed from: http://www.hamiltoncountyhealth.org/files/files/Falls_Issue_Brief.pdf
17. Kaczorowski, J., Chambers, L. W., Karwalajtys, T., Dolovich, L., Farrell, B., McDonough, B., Sebaldt, R., et al. (2008). Cardiovascular Health Awareness Program (CHAP): A community clusterrandomised trial among elderly Canadians. *Preventive medicine*, *46*(6), 537–44.

18. Kaczorowski J, Chambers LW, Dolovich L, Paterson JM, Karwalajtys T, Gierman T et al. (2011) Improving cardiovascular health at population level: 39 community cluster randomized trial of Cardiovascular Health Awareness Program (CHAP). *BMJ*, 342:d442.
19. Ontario Government (2012). *Ontario's Action Plan for Health Care.* Accessed from: http://www.health.gov.on.ca/en/ms/ecfa/healthy_change/docs/rep_healthychange.pdf
20. Kooner,D.L. (2009). All together now: A conceptual exploration of integrated care. *Health Care Quarterly*, 13:6-15.
21. Armitage G.D.,Suter,E.,Oelke,N.,Adair,C. (2009) Health Systems Integration: state of the evidence. *International Journal of Integrated Care*, *17*(9): e82.
22. Shaw,S. and Rosen R. (2013). Fragmentation: a wicked problem with an integrated solution? *Journal of Health Services Research Policy,18*(1):61-4.
23. MacAdam, M. (2008) *Frameworks of Integrated Care for the Elderly: A Systematic Review*. Accessed from: http://www.insp.mx.libaccess.lib.mcmaster.ca/geriatria/acervo/pdf/60%2049813_EN.pdf
24. MacAdam M., 2008; Coleman K., Austin B., Brach C. and Wagner E. (2013). Evidence on the Chronic Care Model in the new millennium. *Health Affairs, 28*(1): 75-85.
25. Collins, R., Peto, R., MacMahon, S., Hebert, P., Fiebach, N.H., Eberlein, K.A. et al. (1990). Blood pressure, stroke, and coronary heart disease. Part 2, short-term reductions in blood pressure: Overview of randomized drug trials in their epidemiological context. *Lancet, 335*, 827-38.
26. Public Health Agency of Canada (2011). *Diabetes in Canada: Facts and figures from a public health perspective*. Accessed from: http://www.phac-aspc.gc.ca/cd-mc/publications/diabetesdiabete/facts-figures-faits-chiffres-2011/highlights-saillants-eng.php
27. Canadian Diabetes Association (2009). *Canadian DiabetesAssociation 2008 Clinical Practice Guidelines for the prevention and management of diabetes in Canada: Executive Summary.* Accessed from: http://www.diabetes.ca/files/for-professionals/CPGExecSummaryEssentials.pdf
28. Harris, D.E., Hamel, L., Aboueissa, A., & Johnson, D. (2011). A cardiovascular disease risk factor screening program designed to reach rural residents of Maine, USA. Rural and Remote Health, 11. Accessed from: http://www.rrh.org.au/articles/subviewnew.asp?ArticleID=1727
29. Wee, L.E., Koh, G.C., Yeo, W.X., Chin, R.T., Wong, J., & Seow, G. (2012). Screening for cardiovascular disease risk factors in an urban low-income setting at baseline and post intervention: A prospective intervention study. *European Journal of Preventive Cardiology,* 1-13.
30. Choi, M & Hector, M. (2012). Effectiveness of Intervention Programs In Preventing Falls: A Systematic Review of Recent 10 Years and Meta-Analysis. *JAMDA, 13 (2),* 188e13-e12.
31. Elley, C.R., Robertson, M.C., Kerse, N.M., Garrett, S., McKinlay, E., Lawton, B., et al. (2007). Falls assessment clinical trial (FACT): Design, interventions, recruitment strategies and participant characteristics. *BMC Public Health, 7.*
32. Tinetti, M.E. (2003). Preventing falls in elderly persons. *NEJM, 348,* 42-49.
33. Gibler, K. (2003). Aging subsidized housing residents: A growing problem in U.S. cities. *Journal of Real Estate Research, 22,* 395-420.
34. Sanders, A., & Stone, R. (2011). *Supporting Aging in Place in Subsidized Housing: An Evaluation of the Well Elder Program.* Accessed from: http://leadingage.org/uploadedFiles/Content/About/Center_for_Applied_Research/Expanding_Affor dable_Housing_Plus_Services/WellElder_Evaluation_Final_Report.pdf.
35. Emergency Medical Services Ontario (2013). *A survey of community paramedicine programs*. Accessed from:

https://emsontario.ca/cms/images/Community%20Paramedics,%20ON.%20Survey%20-%20April%209,%202013.pdf

36. Bigham, B.L., Kennedy, S.B., Drennan, I., Morrison, L.J. (2013). Expanding Paramedic Scope of Practice in the Community: A Systematic Review of the Literature. *Prehospital Emergency Care, 17*: 361-72.
37. Weiss, S.J., Ernst, A.A., Phillips, J., & Hill, B. (2001). Visits to the home environments by emergency medical services: A statewide study. *Prehosp Emerg Care, 5,* 19-22.
38. Weiss, S.J., Chong, R., Ong, M., Ernst, A.A., & Balash, M. (2003). Emergency medical services screening of elderly falls in the home. *Prehosp Emerg Care, 7,* 79-84.
39. Wilber, K.H., Allen, D., Shannon, G.R., Alongi, S. (2003). Partnering managed care and community-based services for frail elders: The care advocate program. *J Am Geriatr Soc, 53,* 80712.
40. Kue, R., Ramstrom, E., Weisberg, S., & Restuccia, M. (2009). Evaluation of an emergency medical services-based social services referral program for elderly patients. *Prehosp Emerg Care, 13,* 27379.
41. Mason, S., Wardrops, J., & Perrin, J. (2003). Developing a community paramedic practitioner intermediate care support scheme for older people with minor conditions. *Emerg Med J, 20,* 196-98.
42. Bronskill, S.E., Camacho, X., Corbett, L., Gill, S.S., Grunier, A., Ho, M., et al. (2011). *Health Systems Unse by frail Ontario seniors.* Accessed from: http://www.ices.on.ca/file/ICES_AgingReport_2011.pdf
43. Bronskill, S.E., Carter, M.W., Costa, A. P., Esensoy, A.V., Gill, S.S., Grunier, A., et al. (2010). *Aging in Ontario: An ICES chartbook of health services used by older adults*. Accessed from: http://www.ices.on.ca/file/AAH%20Chartbook_interactive_final_Feb2010.pdf.
44. Reeve, C., Pashen, D., Mumme, H., De la Rue, S., & Cheffins, T. (2008). Expanding the role of paramedics in northern Queensland: an evaluation of population health training. *The Australian Journal of Rural Health*, *16*(6), 370–5.
45. Stirling, C. M., O'Meara, P., Pedler, D., Tourle, V., & Walker, J. (2007). Engaging rural communities in health care through a paramedic expanded scope of practice. *Rural and Remote Health*, *7*(4), 839.
46. O'Meara, P. F., Tourle, V., Stirling, C., Walker, J., & Pedler, D. (2012). Extending the paramedic role in rural Australia: a story of flexibility and innovation. *Rural and remote health*, *12*(2), 1978.
47. Mason, S., Knowles, E., Colwell, B., Dixon, S., Wardrope, J., Gorringe, R., Snooks, H., et al. (2007). Effectiveness of paramedic practitioners in attending 999 calls from elderly people in the community: cluster randomised controlled trial. *BMJ (Clinical research ed.)*, *335*(7626), 919.
48. Agarwal, G., Kaczorowski, J., Gerstein, H., Hanna S. (2009, November). *Effectiveness of a community-based diabetes program to increase awareness and detection of diabetes.* Paper presented at the annual meeting of the North American Primary Care Research Group (NAPCRG), Montreal, Quebec, Canada.
49. Robinson, C.A., Agarwal, G., & Neremberg, K. (2011). Validating the CANRISK prognostic model for assessing diabetes risk in Canada's multi-ethnic population. *Chronic Dis Inj Can, 32*(1). 19-31.
50. Nordin, E., Lindelöf, N., Rosendahl, E., Jensen, J., & Lundin-Olsson, L. (2008). Prognostic validity of the Timed Up-and-Go test, a modified Get-Up-and-Go test, staff's global judgement and fall history in evaluating fall risk in residential care facilities. *Age and ageing*, *37*(4), 442–8.

51. Beauchet, O., Fantino, B., Allali, G., Muir, S. W., & Annweiler, C. (2011). Timed up and go test and risk of falls in older adults: A systematic review. *The Journal of Nutrition, Health, & Aging*, *15*(10), 6–11.
52. Agarwal, G. (2011). *Primary Care Diabetes: Issues facting Family Doctors. Open Access Dissertations and Theses.* Paper 6432.http://digitalcommons.mcmaster.ca/opendissertations/6432
53. Creswell, John (2007). *Qualitative Inquiry & Research Design: Choosing Among Five Approaches*. Thousand Oaks, CA: Sage Publications, Inc. pp. 178-180.
54. Statistics Canada. *Canadian demographics at a glance.* Accessed from: http://www.statcan.gc.ca/pub/91-003-x/91-003-x2007001-eng.pdf
