# Supplementary material for: Community Paramedicine Program in Social Housing and Health Service Utilization: A Cluster Randomized Clinical Trial
Source: JAMA Netw Open. 2024 Oct 28;7(10):e2441288. doi: 10.1001/jamanetworkopen.2024.41288 (PMC11581518; doi:10.1001/jamanetworkopen.2024.41288)
Supplement: Supplement 2. — eTable 1. Disease and Procedure Codes eTable 2. Medication Categories eTable 3. Participant Characteristics of Intervention Building Residents, Control Building Residents, and Intervention Attendees Who Had Prescription Drug Claim Records Available (≥66 Years or Eligible for the Ontario Drug Benefit) eTable 4. Intention-to-Treat Analysis of Medication Initiation for All Intervention and Control Building Residents eTable 5. Sensitivity Analysis of Healthcare Utilization and Medication Initiation Outcomes of Building Residents That Attended the Intervention Program eTable 6. Sensitivity Analysis of Healthcare Utilization and Medication Initiation Outcomes of All Building Residents for a Subset of Buildings (N = 26) eTable 7. Sensitivity Analysis of Healthcare Utilization and Medication Initiation Outcomes of Building Residents That Attended the Intervention Program for a Subset of Buildings (N = 26) [file jamanetwopen-e2441288-s002.pdf]

Supplementary Online Content

Agarwal G, Pirrie M, Angeles R, et al. Community paramedicine program in social housing and health service utilization: a cluster randomized clinical trial. *JAMA Netw Open*. 2024;7(10):e2441288. doi:10.1001/jamanetworkopen.2024.41288

eTable 1. Disease and Procedure Codes

eTable 2. Medication Categories

eTable 3. Participant Characteristics of Intervention Building Residents, Control Building Residents, and Intervention Attendees Who Had Prescription Drug Claim Records Available (≥ 66 Years or Eligible for the Ontario Drug Benefit)

eTable 4. Intention-to-Treat Analysis of Medication Initiation for All Intervention and Control Building Residents

eTable 5. Sensitivity Analysis of Healthcare Utilization and Medication Initiation Outcomes of Building Residents That Attended the Intervention Program

eTable 6. Sensitivity Analysis of Healthcare Utilization and Medication Initiation Outcomes of All Building Residents for a Subset of Buildings (N=26)

eTable 7. Sensitivity Analysis of Healthcare Utilization and Medication Initiation Outcomes of Building Residents That Attended the Intervention Program for a Subset of Buildings (N=26)

This supplementary material has been provided by the authors to give readers additional information about their work.

© 2024 Agarwal G et al. *JAMA Network Open.*

eTable 1. Disease and Procedure Codes

**ICD10 codes for cardiovascular disease and peripheral vascular disease**
NACRS AND DAD DIAGNOSIS CODES (DXTYPE 1 or 2, excluding suspect)<br><br>AMI: 'I210', 'I211', 'I212', 'I213', 'I214', 'I219', 'I220', 'I221' , 'I228', ‘I229', 'I230', 'I231', 'I232', 'I233', 'I235', 'I236', 'I238', 'I241', 'I252', 'I256'<br><br>Stroke: 'I60', 'I61', 'I630', 'I631', 'I632', 'I633', 'I634', 'I635', 'I636', 'I638', 'I639', 'I64', 'H341'<br><br>CHD/PVD: I25x, I70x, I71x, I73x, I74x, K55.1
SDS AND DAD PROCEDURE CODES<br><br>PCI: CCI 1IJ50, 1IJ57GQ<br><br>CABG: CCI 1IJ76<br><br>Carotid endarterectomy: CCI 1JE57
**Lab Testing Codes**
KIDNEY<br><br>• L067 - LAB. MED.-BIOCHEM-CREATININE (NOT WITH L068).
ELECTROLYTE<br><br>• L204 - LAB. MED.-BIOCHEM.-POTASSIUM<br>• L226 - LAB. MED.-BIOCHEM.-SODIUM<br>• L251 - LAB.MED.-BIOCHEM.-UREA NITROGEN (B.U.N.)
LIPIDS<br><br>• L055 - LAB. MED.-BIOCHEM-CHOLESTROL,TOTAL (NOT WITH L156).<br>• L117 - LAB. MED.-BIOCHEM.-HIGH DENSITY LIPOPROTEIN CHOLESTEROL<br>• L151 - LAB.MED-BIOCHEM.LIPID TOTAL
DIABETES<br><br>• L093 - LAB. MED.-BIOCHEM. GLYCOSYLATED HAEMOGLOBIN<br>• L104 - LAB. MED.-BIOCHEM.-GLUCOSE TOLERANCE TEST

© 2024 Agarwal G et al. *JAMA Network Open.*

eTable 2. Medication Categories

Category	Drug Name
ANTIHYPERTENSIVE	ACEBUTOLOL
ANTIHYPERTENSIVE	ALISKIREN
ANTIHYPERTENSIVE	AMILORIDE HCL
ANTIHYPERTENSIVE	AMILORIDE HCL & HYDROCHLOROTHIAZIDE
ANTIHYPERTENSIVE	AMLODIPINE BESYLATE
ANTIHYPERTENSIVE	AMLODIPINE BESYLATE & ATORVASTATIN CALCIUM
ANTIHYPERTENSIVE	AMLODIPINE BESYLATE & TELMISARTAN
ANTIHYPERTENSIVE	ATENOLOL
ANTIHYPERTENSIVE	ATENOLOL & CHLORTHALIDONE
ANTIHYPERTENSIVE	BENAZEPRIL CHLOROHYDRATE
ANTIHYPERTENSIVE	BENAZEPRIL
ANTIHYPERTENSIVE	BISOPROLOL FUMARATE
ANTIHYPERTENSIVE	CANDESARTAN CILEXETIL
ANTIHYPERTENSIVE	CANDESARTAN CILEXETIL & HYDROCHLOROTHIAZIDE
ANTIHYPERTENSIVE	CAPTOPRIL
ANTIHYPERTENSIVE	CARVEDILOL
ANTIHYPERTENSIVE	CHLORTHALIDONE
ANTIHYPERTENSIVE	CILAZAPRIL
ANTIHYPERTENSIVE	CILAZAPRIL & HYDROCHLOROTHIAZIDE
ANTIHYPERTENSIVE	DILTIAZEM
ANTIHYPERTENSIVE	ENALAPRIL MALEATE
ANTIHYPERTENSIVE	ENALAPRIL SODIUM
ANTIHYPERTENSIVE	EPLERENONE
ANTIHYPERTENSIVE	EPROSARTAN MESYLATE
ANTIHYPERTENSIVE	EPROSARTAN MESYLATE & HYDROCHLOROTHIAZIDE
ANTIHYPERTENSIVE	FELODIPINE
ANTIHYPERTENSIVE	FOSINOPRIL SODIUM
ANTIHYPERTENSIVE	FUROSEMIDE
ANTIHYPERTENSIVE	HYDROCHLOROTHIAZIDE
ANTIHYPERTENSIVE	HYDROCHLOROTHIAZIDE & IRBESARTAN
ANTIHYPERTENSIVE	HYDROCHLOROTHIAZIDE & LISINOPRIL
ANTIHYPERTENSIVE	HYDROCHLOROTHIAZIDE & LOSARTAN POTASSIUM
ANTIHYPERTENSIVE	HYDROCHLOROTHIAZIDE & METHYLDOPA
ANTIHYPERTENSIVE	HYDROCHLOROTHIAZIDE & OLMESARTAN MEDOXOMIL
ANTIHYPERTENSIVE	HYDROCHLOROTHIAZIDE & PINDOLOL
ANTIHYPERTENSIVE	HYDROCHLOROTHIAZIDE & PROPRANOLOL HCL
ANTIHYPERTENSIVE	HYDROCHLOROTHIAZIDE & QUINAPRIL
ANTIHYPERTENSIVE	HYDROCHLOROTHIAZIDE & RAMIPRIL
ANTIHYPERTENSIVE	HYDROCHLOROTHIAZIDE & SPIRONOLACTONE
ANTIHYPERTENSIVE	HYDROCHLOROTHIAZIDE & TELMISARTAN
ANTIHYPERTENSIVE	HYDROCHLOROTHIAZIDE & TIMOLOL MALEATE

© 2024 Agarwal G et al. *JAMA Network Open.*

ANTIHYPERTENSIVE	HYDROCHLOROTHIAZIDE & TRIAMTERENE
ANTIHYPERTENSIVE	HYDROCHLOROTHIAZIDE & VALSARTAN
ANTIHYPERTENSIVE	INDAPAMIDE
ANTIHYPERTENSIVE	INDAPAMIDE & PERINDOPRIL TERT.BUTYLAMINE
ANTIHYPERTENSIVE	IRBESARTAN
ANTIHYPERTENSIVE	LABETALOL HCL
ANTIHYPERTENSIVE	LISINOPRIL
ANTIHYPERTENSIVE	LOSARTAN POTASSIUM
ANTIHYPERTENSIVE	METOLAZONE
ANTIHYPERTENSIVE	METOPROLOL
ANTIHYPERTENSIVE	METOPROLOL SUCCINATE
ANTIHYPERTENSIVE	METOPROLOL SULFATE
ANTIHYPERTENSIVE	METOPROLOL TARTRATE
ANTIHYPERTENSIVE	NADOLOL
ANTIHYPERTENSIVE	NICARDIPINE HCL
ANTIHYPERTENSIVE	NIFEDIPINE
ANTIHYPERTENSIVE	OLMESARTAN MEDOXOMIL
ANTIHYPERTENSIVE	OXPRENOLOL HCL
ANTIHYPERTENSIVE	PERINDOPRIL TERT.BUTYLAMINE
ANTIHYPERTENSIVE	PINDOLOL
ANTIHYPERTENSIVE	PROPIVERINE HCL
ANTIHYPERTENSIVE	PROPRANOLOL HCL
ANTIHYPERTENSIVE	QUINAPRIL
ANTIHYPERTENSIVE	RAMIPRIL
ANTIHYPERTENSIVE	SOTALOL
ANTIHYPERTENSIVE	SPIRONOLACTONE
ANTIHYPERTENSIVE	TELMISARTAN
ANTIHYPERTENSIVE	TIMOLOL
ANTIHYPERTENSIVE	TIMOLOL MALEATE
ANTIHYPERTENSIVE	TRANDOLAPRIL
ANTIHYPERTENSIVE	TRIAMTERENE
ANTIHYPERTENSIVE	VALSARTAN
ANTIHYPERTENSIVE	VERAPAMIL HCL
ANTI-PLATELET/ANTICOAGULANT	ACENOCOUMAROL
ANTI-PLATELET/ANTICOAGULANT	ACETYLSALICYLIC ACID & DIPYRIDAMOLE
ANTI-PLATELET/ANTICOAGULANT	ANAGRELIDE HCL
ANTI-PLATELET/ANTICOAGULANT	APIXABAN
ANTI-PLATELET/ANTICOAGULANT	CLOPIDOGREL
ANTI-PLATELET/ANTICOAGULANT	CLOPIDOGREL BISULFATE
ANTI-PLATELET/ANTICOAGULANT	DABIGATRAN ETEXILATE
ANTI-PLATELET/ANTICOAGULANT	DIPYRIDAMOLE
ANTI-PLATELET/ANTICOAGULANT	EDOXABAN TOSYLATE
ANTI-PLATELET/ANTICOAGULANT	PRASUGREL HCL
ANTI-PLATELET/ANTICOAGULANT	RIVAROXABAN
ANTI-PLATELET/ANTICOAGULANT	TICAGRELOR
ANTI-PLATELET/ANTICOAGULANT	TICLOPIDINE
ANTI-PLATELET/ANTICOAGULANT	WARFARIN SODIUM
DIABETES	ACARBOSE
DIABETES	CANAGLIFLOZIN
DIABETES	CHLORPROPAMIDE
DIABETES	DAPAGLIFLOZIN
DIABETES	DAPAGLIFLOZIN & METFORMIN HCL
DIABETES	EMPAGLIFLOZIN

DIABETES	EMPAGLIFLOZIN & METFORMIN HCL
DIABETES	GLICLAZIDE
DIABETES	GLIMEPIRIDE
DIABETES	GLYBURIDE
DIABETES	HUMAN INSULIN ISOPHANE RECOMBINANT
DIABETES	HUMAN INSULIN RECOMBINANT
DIABETES	HUMAN INSULIN RECOMBINANT & HUMAN INSULIN ISOPHANE RECOMBINANT
DIABETES	HUMAN INSULIN ZINC RECOMBINANT
DIABETES	INSULIN
DIABETES	INSULIN & INSULIN ISOPHANE
DIABETES	INSULIN (ZINC CRYSTALLINE) HUMAN BIOSYNTHETIC (RDNA ORIGIN)
DIABETES	INSULIN (ZINC) BEEF
DIABETES	INSULIN ASPART RECOMBINANT
DIABETES	INSULIN DEGLUDEC
DIABETES	INSULIN DEGLUDEC
DIABETES	INSULIN DETEMIR RECOMBINANT
DIABETES	INSULIN GLARGINE RECOMBINANT
DIABETES	INSULIN GLULISINE RECOMBINANT
DIABETES	INSULIN ISOPHANE
DIABETES	INSULIN LISPRO RECOMBINANT
DIABETES	INSULIN LISPRO RECOMBINANT & INSULIN LISPRO PROTAMINE RECOMBINANT
DIABETES	INSULIN PORCINE BASE
DIABETES	INSULIN PORCINE BASE ISOPHANE
DIABETES	INSULIN PROTAMINE ZINC
DIABETES	INSULIN ZINC
DIABETES	LINAGLIPTIN
DIABETES	LINAGLIPTIN & METFORMIN HCL
DIABETES	METFORMIN HCL
DIABETES	METFORMIN HCL & SAXAGLIPTIN
DIABETES	METFORMIN HCL & SITAGLIPTIN PHOSPHATE
DIABETES	NATEGLINIDE
DIABETES	PIOGLITAZONE HCL
DIABETES	REPAGLINIDE
DIABETES	ROSIGLITAZONE MALEATE
DIABETES	SAXAGLIPTIN HCL
DIABETES	SITAGLIPTIN PHOSPHATE
DIABETES	TOLBUTAMIDE
LIPID-LOWERING	ATORVASTATIN CALCIUM
LIPID-LOWERING	BEZAFIBRATE
LIPID-LOWERING	CERIVASTATIN SODIUM
LIPID-LOWERING	CLOFIBRATE
LIPID-LOWERING	FENOFIBRATE
LIPID-LOWERING	FLUVASTATIN
LIPID-LOWERING	FLUVASTATIN SODIUM
LIPID-LOWERING	GEMFIBROZIL
LIPID-LOWERING	LOVASTATIN
LIPID-LOWERING	PRAVASTATIN
LIPID-LOWERING	PRAVASTATIN SODIUM
LIPID-LOWERING	ROSUVASTATIN CALCIUM
LIPID-LOWERING	SIMVASTATIN

© 2024 Agarwal G et al. *JAMA Network Open.*

eTable 3: Participant characteristics of intervention building residents, control building residents, and intervention attendees who had prescription drug claim records available (≥ 66 years or eligible for the Ontario Drug Benefit)

	**All buildings** (N = 30)			**Subset of buildings** (N = 26)		
Demographic information	**Control** All residents n = 1,721	**Intervention** All residents n = 1,761	**Intervention** Attendees only n = 399	**Control** All residents n = 1,388	**Intervention** All residents n = 1,311	**Intervention** Attendees only n = 355
Age (years)						
Mean ± SD	73.23 ± 8.80	73.60 ± 8.95	73.90 ± 8.79	74.29 ± 8.68	74.11 ± 8.23	74.36 ± 8.43
Median ± IQR	73 (66-79)	73 (67-80)	74 (67-80)	74 (68-80)	74 (68-80)	75 (68-80)
Sex						
Female	1,079 (62.7%)	1,208 (68.6%)	332 (83.2%)	913 (65.8%)	954 (72.8%)	298 (83.9%)
Male	642 (37.3%)	553 (31.4%)	67 (16.8%)	475 (34.2%)	357 (27.2%)	57 (16.1%)

© 2024 Agarwal G et al. *JAMA Network Open.*

eTable 4. Intention-to-Treat Analysis of Medication Initiation for All Intervention and Control Building Residents

Medication category	No./total No. (%) of residents				AOR (95% CI)[a]
	Baseline prevalence (N = 30 buildings)[b]		Initiation of new medication (N = 30 buildings)[b,c]		
	Control	Intervention	Control	Intervention	
Diabetes	418/1721 (24.3)	437/1761 (24.8%)	22/1303 (1.7)	28/1324 (2.1)	1.25 (0.68-2.30)
Antihypertensives	1169/1721 (67.9)	1261/1761 (71.6%)	47/552 (8.5)	74/500 (14.8)	1.74 (1.19-2.53)d
Anticoagulants	279/1721 (16.2)	281/1761 (16.0%)	69/1442 (4.8)	48/1481 (3.2%)	0.68 (0.53-0.86)d
Statins or fibrates	918/1721 (53.3)	926/1761 (52.6%)	62/803 (7.7)	63/835 (7.5%)	0.98 (0.83-1.16)

Abbreviation: AOR, adjusted odds ratio.

[a]Adjusted for clustering of residents within buildings and the building pairing (trial design).

[b]Medication baseline prevalence and initiation were only available for individuals with a medication record in the administrative data (ie, eligible for the Ontario Drug Benefit).

[c]Medication initiation was restricted to individuals who had not taken a medication in that category for at least 12 months before the intervention.

[d]Statistically significant at $P < .05$.

© 2024 Agarwal G et al. *JAMA Network Open.*

eTable 5: Sensitivity analysis of healthcare utilization and medication initiation outcomes of building residents that attended the intervention program

	**Baseline** (N = 30 Buildings)		**Post-Intervention** (N = 30 Buildings)		
**Binary outcomes**	**Control**<br>All residents<br>n = 1,846	**Intervention**<br>Attendees only<br>n = 412	**Control**<br>All residents<br>n = 1,846	**Intervention**<br>Attendees only<br>n = 412	**Adjusted Odds Ratio[a] [95%CI]**
ED visits	689 (37.3%)	184 (44.7%)	764 (41.4%)	206 (50.0%)	1.10 [0.99,1.22]
ED visits by ambulance	368 (19.9%)	91 (22.1%)	463 (25.1%)	121 (29.4%)	1.09 [0.99,1.21]
Hospital admissions	264 (14.3%)	64 (15.5%)	338 (18.3%)	79 (19.2%)	1.02 [0.89,1.18]
Home care services	430 (23.3%)	118 (28.6%)	499 (27.0%)	146 (35.4%)	**1.07 [1.01,1.13]**
Transfers to long term care	--------	--------	83 (4.5%)	6 (1.5%)	**0.32 [0.13,0.81]**
Kidney function lab test	1,229 (66.6%)	317 (76.9%)	1,277 (69.2%)	323 (78.4%)	1.04 [0.98,1.10]
Electrolyte lab test	1,071 (58.0%)	276 (67.0%)	1,095 (59.3%)	278 (67.5%)	1.06 [0.97,1.15]
Lipids lab test	1,011 (54.8%)	257 (62.4%)	999 (54.1%)	246 (59.7%)	1.02 [0.95,1.11]
Diabetes lab test	946 (51.2%)	248 (60.2%)	1,000 (54.2%)	262 (63.6%)	1.06 [0.99,1.14]
**Medication[b]**	**Baseline prevalence**		**Initiation of new medication**		
	n = 1,721	n = 399	n = 1,303; 552; 1,442; 803[b]	n = 293; 98; 322; 179[b]	
Diabetes	418 (24.3%)	106 (26.6%)	22 (1.7%)	7 (2.4%)	1.42 [0.67, 3.00]
Antihypertensives	1,169 (67.9%)	301 (75.4%)	47 (8.5%)	23 (23.5%)	**2.76 [1.80, 4.22]**
Anticoagulants	279 (16.2%)	77 (19.3%)	69 (4.8%)	9 (2.8%)	0.58 [0.28, 1.20]
Statins/fibrates	918 (53.3%)	220 (55.1%)	62 (7.7%)	12 (6.7%)	0.87 [0.41, 1.83]
**Continuous outcomes**	**Control**<br>All residents	**Intervention**<br>Attendees only	**Control**<br>All residents	**Intervention**<br>Attendees only	**Adjusted Incidence Rate Ratio[a] [95%CI]**
Primary care visits	n = 1,846	n = 412	n = 1,846	n = 412	
Mean ± SD	5.94 ± 6.59	6.56 ± 6.54	6.29 ± 6.77	7.02 ± 6.08	**1.10 [1.03,1.17]**
Hospitalization length of stay[c]	n = 264	n = 64	n = 338	n = 79	
Mean ± SD	13.05 ± 21.89	9.55 ± 10.53	18.78 ± 37.20	12.99 ± 19.54	0.69 [0.46,1.04]

Notes: [a] Except for the 'transfers to long term care' and 'initiation of new medication' outcomes, all odds ratios and incident rate ratios were calculated using generalized estimated equation (GEE) models adjusted for clustering of residents within buildings, building pairing (trial design), and the baseline value. For the 'transfers to long term care' and 'initiation of new medication' outcomes, the GEE model was adjusted for clustering of residents within buildings and the building pairing only. [b] Medication initiation was restricted to individuals who had not taken a medication in that category for at least 12 months prior to the intervention and where a medication record was available in the administrative data (i.e., eligible for the Ontario Drug Benefit [ODB]).[c] The 'hospitalization length of stay' outcome was restricted to only those who had been hospitalized.

© 2024 Agarwal G et al. *JAMA Network Open.*

eTable 6: Sensitivity analysis of healthcare utilization and medication initiation outcomes of all building residents for a subset of buildings (N=26)

	**Baseline** (N = 26 Buildings)		**Post-Intervention** (N = 26 Buildings)		
**Binary outcomes**	**Control** All residents n = 1,468	**Intervention** All residents n = 1,358	**Control** All residents n = 1,468	**Intervention** All residents n = 1,358	**Adjusted Odds Ratio[a] [95%CI]**
ED visits	551 (37.5%)	514 (37.8%)	616 (42.0%)	563 (41.5%)	0.98 [0.90,1.06]
ED visits by ambulance	298 (20.3%)	239 (17.6%)	370 (25.2%)	306 (22.5%)	0.93 [0.83,1.04]
Hospital admissions	212 (14.4%)	180 (13.3%)	277 (18.9%)	232 (17.1%)	0.94 [0.84,1.05]
Home care services	352 (24.0%)	294 (21.6%)	409 (27.9%)	361 (26.6%)	1.00 [0.91,1.11]
Transfers to long term care	--------	--------	73 (5.0%)	55 (4.1%)	0.81 [0.62,1.08]
Kidney function lab test	1,009 (68.7%)	996 (73.3%)	1,045 (71.2%)	1,004 (73.9%)	0.99 [0.95,1.04]
Electrolyte lab test	887 (60.4%)	858 (63.2%)	909 (61.9%)	870 (64.1%)	1.00 [0.95,1.05]
Lipids lab test	831 (56.6%)	813 (59.9%)	812 (55.3%)	814 (59.9%)	1.04 [0.98,1.10]
Diabetes lab test	784 (53.4%)	751 (55.3%)	817 (55.7%)	798 (58.8%)	1.03 [0.98,1.07]
**Medication[b]**	**Baseline prevalence**		**Initiation of new medication**		
	n = 1,388	n = 1,311	n = 1,044; 414; 1,146; 632[b]	n = 988; 359; 1,094; 617[b]	
Diabetes	344 (24.8%)	323 (24.6%)	17 (1.6%)	24 (2.4%)	1.49 [0.74, 3.01]
Antihypertensives	974 (70.2%)	952 (72.6%)	35 (8.5%)	61 (17.0%)	**2.01 [1.33, 3.04]**
Anticoagulants	242 (17.4%)	217 (16.6%)	57 (5.0%)	35 (3.2%)	**0.64 [0.48, 0.86]**
Statins/fibrates	756 (54.5%)	694 (52.9%)	49 (7.8%)	48 (7.8%)	1.00 [0.81, 1.24]
**Continuous outcomes**	**Control** All residents	**Intervention** All residents	**Control** All residents	**Intervention** All residents	**Adjusted Incidence Rate Ratio[a] [95%CI]**
Primary care visits	n = 1,468	n = 1,358	n = 1,468	n = 1,358	
Mean ± SD	5.94 ± 6.40	6.42 ± 6.66	6.28 ± 6.61	6.63 ± 6.80	1.01 [0.95,1.08]
Hospitalization length of stay[c]	n = 212	n = 180	n = 277	n = 232	
Mean ± SD	12.89 ± 22.84	11.10 ± 19.90	17.08 ± 28.17	16.01 ± 23.25	0.94 [0.80, 1.11]

Notes: [a] Except for the 'transfers to long term care' and 'initiation of new medication' outcomes, all odds ratios and incident rate ratios were calculated using generalized estimated equation (GEE) models adjusted for clustering of residents within buildings, building pairing (trial design), and the baseline value. For the 'transfers to long term care' and 'initiation of new medication' outcomes, the GEE model was adjusted for clustering of residents within buildings and the building pairing only. [b] Medication initiation was restricted to individuals who had not taken a medication in that category for at least 12 months prior to the intervention and where a medication record was available in the administrative data (i.e., eligible for the Ontario Drug Benefit [ODB]).[c] The 'hospitalization length of stay' outcome was restricted to only those who had been hospitalized.

© 2024 Agarwal G et al. *JAMA Network Open.*

eTable 7: Sensitivity analysis of healthcare utilization and medication initiation outcomes of building residents that attended the intervention program for a subset of buildings (N=26)

	**Baseline** (N = 26 Buildings)		**Post-Intervention** (N = 26 Buildings)		
**Binary outcomes**	**Control**<br>All residents<br>n = 1,468	**Intervention**<br>Attendees only<br>n = 362	**Control**<br>All residents<br>n = 1,468	**Intervention**<br>Attendees only<br>n = 362	**Adjusted Odds Ratio[a] [95%CI]**
ED visits	551 (37.5%)	161 (44.5%)	616 (42.0%)	181 (50.0%)	1.09 [0.97,1.23]
ED visits by ambulance	298 (20.3%)	77 (21.3%)	370 (25.2%)	104 (28.7%)	1.09 [0.96,1.23]
Hospital admissions	212 (14.4%)	54 (14.9%)	277 (18.9%)	70 (19.3%)	1.01 [0.85,1.19]
Home care services	352 (24.0%)	97 (26.8%)	409 (27.9%)	125 (34.5%)	**1.08 [1.01,1.15]**
Transfers to long term care	--------	--------	73 (5.0%)	6 (1.7%)	**0.33 [0.13, 0.85]**
Kidney function lab test	1,009 (68.7%)	284 (78.5%)	1,045 (71.2%)	292 (80.7%)	1.04 [0.98,1.11]
Electrolyte lab test	887 (60.4%)	249 (68.8%)	909 (61.9%)	253 (69.9%)	1.05 [0.95,1.15]
Lipids lab test	831 (56.6%)	229 (63.3%)	812 (55.3%)	218 (60.2%)	1.02 [0.95,1.10]
Diabetes lab test	784 (53.4%)	220 (60.8%)	817 (55.7%)	231 (63.8%)	1.06 [0.98,1.14]
**Medication[b]**	**Baseline prevalence**		**Initiation of new medication**		
	n = 1,388	n = 355	n = 1,044; 414; 1,146; 632[b]	n = 268; 87; 284; 164[b]	
Diabetes	344 (24.8%)	87 (24.5%)	17 (1.6%)	7 (2.6%)	1.60 [0.72, 3.57]
Antihypertensives	974 (70.2%)	268 (75.5%)	35 (8.5%)	22 (25.3%)	**2.99 [1.94, 4.60]**
Anticoagulants	242 (17.4%)	71 (20.0%)	57 (5.0%)	8 (2.3%)	0.57 [0.25, 1.28]
Statins/fibrates	756 (54.5%)	191 (53.8%)	49 (7.8%)	12 (7.3%)	0.94 [0.44, 2.00]
**Continuous outcomes**	**Control**<br>All residents	**Intervention**<br>Attendees only	**Control**<br>All residents	**Intervention**<br>Attendees only	**Adjusted Incidence Rate Ratio[a] [95%CI]**
Primary care visits	n = 1,468	n = 362	n = 1,468	n = 362	
Mean ± SD	5.94 ± 6.40	6.74 ± 6.74	6.28 ± 6.61	7.22 ± 6.21	**1.10 [1.04,1.19]**
Hospitalization length of stay[c]	n = 212	n = 54	n = 277	n = 70	
Mean ± SD	12.89 ± 22.84	10.26 ± 11.19	17.08 ± 28.17	11.91 ± 13.31	**0.69 [0.49, 0.98]**

Notes: [a] Except for the ‘transfers to long term care’ and ‘initiation of new medication’ outcomes, all odds ratios and incident rate ratios were calculated using generalized estimated equation (GEE) models adjusted for clustering of residents within buildings, building pairing (trial design), and the baseline value. For the ‘transfers to long term care’ and ‘initiation of new medication’ outcomes, the GEE model was adjusted for clustering of residents within buildings and the building pairing only. [b] Medication initiation was restricted to individuals who had not taken a medication in that category for at least 12 months prior to the intervention and where a medication record was available in the administrative data (i.e., eligible for the Ontario Drug Benefit [ODB]).[c] The ‘hospitalization length of stay’ outcome was restricted to only those who had been hospitalized.

© 2024 Agarwal G et al. *JAMA Network Open.*
