# Supplementary material for: Community Paramedicine Program in Social Housing and Health Service Utilization: A Cluster Randomized Clinical Trial
Source: JAMA Netw Open. 2024 Oct 28;7(10):e2441288. doi: 10.1001/jamanetworkopen.2024.41288 (PMC11581518; doi:10.1001/jamanetworkopen.2024.41288)
Supplement: Supplement 3. — Data Sharing Statement [file jamanetwopen-e2441288-s003.pdf]

Data Sharing Statement

Agarwal. Community Paramedicine Program in Social Housing and Health Service Utilization. *JAMA Netw Open*. Published October 28, 2024. doi:10.1001/jamanetworkopen.2024.41288

Data
Additional Information: Clinicaltrials.gov, Clinicaltrials.gov, NCT02152891
Data available: Yes
Data types: Deidentified participant data
How to access data: The dataset from this study is held securely in coded form at ICES. Although legal data sharing agreements between ICES and data providers (e.g., healthcare organisations and governments) prohibit ICES from making the dataset publicly available, access may be granted to those who meet prespecified criteria for confidential access, available at https://www.ices.on.ca/DAS (das@ices.on.ca). The full dataset creation plan and underlying analytical code are available from the authors upon request, understanding that the computer programs may rely upon coding templates or macros that are unique to ICES and are therefore either inaccessible or may require modification.
When available: With publication

Supporting Documents
Document types: None

Additional Information
Who can access the data: The dataset from this study is held securely in coded form at ICES. Although legal data sharing agreements between ICES and data providers (e.g., healthcare organisations and governments) prohibit ICES from making the dataset publicly available, access may be granted to those who meet prespecified criteria for confidential access, available at https://www.ices.on.ca/DAS (das@ices.on.ca). The full dataset creation plan and underlying analytical code are available from the authors upon request, understanding that the computer programs may rely upon coding templates or macros that are unique to ICES and are therefore either inaccessible or may require modification.
Types of analyses: The dataset from this study is held securely in coded form at ICES. Although legal data sharing agreements between ICES and data providers (e.g., healthcare organisations and governments) prohibit ICES from making the dataset publicly available, access may be granted to those who meet prespecified criteria for confidential access, available at https://www.ices.on.ca/DAS (das@ices.on.ca). The full dataset creation plan and underlying analytical code are available from the authors upon request, understanding that the computer programs may rely upon coding templates or macros that are unique to ICES and are therefore either inaccessible or may require modification.
Mechanisms of data availability: The dataset from this study is held securely in coded form at ICES. Although legal data sharing agreements between ICES and data providers (e.g., healthcare organisations and governments) prohibit ICES from making the dataset publicly available, access may be granted to those who meet prespecified criteria for confidential access, available at https://www.ices.on.ca/DAS (das@ices.on.ca). The full dataset creation plan and underlying analytical code are available from the authors upon request, understanding that the computer programs may rely upon coding templates or macros that are unique to ICES and are therefore either inaccessible or may require modification.
